# The STAT3–ZEB1 axis contributes to CCL2-mediated resistance to osimertinib in lung cancer

**DOI:** 10.3389/fonc.2026.1699471

**Published:** 2026-02-20

**Authors:** Tzu-Hua Chang, Meng-Feng Tsai, Shang-Gin Wu, Yi-Nan Liu, Huey-Dong Wu, Tzu-Hsiu Tsai, Han-Nian Jheng, Chia-Lang Hsu, Pu-Sheng Yeh, Jin-Yuan Shih

**Affiliations:** 1 Department of Internal Medicine, National Taiwan University Hospital, Taipei, Taiwan; 2 Department of Biomedical Sciences, Da-Yeh University, Changhua, Taiwan; 3 Department of Internal Medicine, National Taiwan University Cancer Center, Taipei, Taiwan; 4 Department of Integrated Diagnostics & Therapeutics, National Taiwan University Hospital, Taipei, Taiwan; 5 Department of Medical Research, National Taiwan University Hospital, Taipei, Taiwan; 6 Department of Critical Care Medicine, Min-Sheng General Hospital, Taoyuan, Taiwan; 7 Graduate Institute of Clinical Medicine, College of Medicine, National Taiwan University, Taipei, Taiwan

**Keywords:** CCL2, epithelial to mesenchymal transition, osimertinib resistance, STAT3, ZEB1

## Abstract

**Objective:**

Osimertinib, a third-generation EGFR tyrosine kinase inhibitor (TKI), is effective in NSCLC patients with EGFR-activating or T790M mutations, but acquired resistance remains a major challenge. Although CCL2 has been implicated in EGFR-TKI resistance via AKT activation, the precise downstream mechanisms are not fully understood.

**Methods:**

We analyzed malignant pleural effusion samples from patients with resistant NSCLC and conducted functional assays in lung cancer cell lines with ectopic CCL2 expression or knockdown, combined with *in vivo* xenograft models. Key downstream signaling pathways were interrogated.

**Results:**

CCL2 was significantly upregulated in resistant patient samples. Overexpression of CCL2 induced osimertinib resistance, whereas silencing restored drug sensitivity. Mechanistically, CCL2 promoted resistance through STAT3- and ERK1/2-dependent upregulation of ZEB1, rather than via the AKT pathway. Notably, combined STAT3 inhibition and osimertinib effectively reversed resistance in xenografts.

**Conclusion:**

These findings uncover a novel CCL2–STAT3–ZEB1 signaling axis that drives acquired osimertinib resistance in NSCLC. Dual targeting of STAT3 and EGFR may represent a promising therapeutic approach to improve clinical outcomes.

## 1 Introduction

Lung cancer remains the leading cause of cancer-related deaths worldwide, with a dismal five-year survival rate of only 10–20% in most countries. Non-small-cell lung cancer (NSCLC) constitutes approximately 85% of all lung cancer cases (1, 2). Unfortunately, conventional treatment options such as surgery, radiation therapy, and platinum-based chemotherapy offer limited efficacy and are associated with poor outcomes (3). Given these challenges, molecular-targeted therapies have emerged as a promising treatment strategy for patients with NSCLC. The epidermal growth factor receptor (EGFR) is a well-established molecular target due to its critical role in tumor initiation and progression (4). Somatic activating mutations in EGFR are detected in approximately 40–50% of patients with non-squamous NSCLC in Asia and 10–20% in Western countries, and these patients have exhibited favorable responses to EGFR-tyrosine kinase inhibitors (EGFR-TKIs) (5). However, a major limitation of first- and second-generation EGFR-TKIs is the inevitable development of acquired resistance, most commonly through the emergence of the T790M mutation, which occurs in approximately 50–60% of cases (6). Osimertinib, a third-generation EGFR-TKI, has demonstrated potent efficacy in patients harboring with T790M resistance mutation (7) as well as in treatment-naïve patients with EGFR-activating mutations (8). Despite the initial positive response, the development of acquired resistance to osimertinib remains a significant clinical obstacle. The mechanisms driving this resistance are heterogeneous and multifactorial, involving both EGFR-dependent pathways (6, 9) and EGFR-independent mechanisms (10, 11). Additional contributors include alterations in alternative tyrosine kinase receptors (12, 13), oncogenic fusions (14, 15), and the induction of epithelial-mesenchymal transition (EMT) (16). Notably, in nearly 50% of NSCLC patients with acquired resistance to osimertinib, the underlying mechanisms remain unknown, underscoring the urgent need for further investigation to elucidate novel pathways involved in resistance and identify new therapeutic strategies.

Chemokine C-C motif ligand 2 (CCL2), also known as monocyte chemoattractant protein-1 (MCP1), belongs to the CC chemokine family (17). It plays a pivotal role in immune cell recruitment, inflammation, and tumor progression (18). Recent investigations have elucidated that CCL2 is involved in mediating drug resistance across various cancer types (19). By modulating the tumor microenvironment, CCL2 promotes the recruitment of immunosuppressive cells, such as tumor-associated macrophages, which facilitate drug resistance through paracrine signaling (20, 21). Moreover, CCL2 has been shown to enhances chemoresistance in cancer cells by elevating its own expression, thereby diminishing the efficacy of chemotherapeutic agents including taxanes, platinum compounds, and temozolomide (22–25). It reduces drug-induced apoptosis by activating the AKT signaling pathway (22, 24) and promoting glycolysis (22), thus enhancing cancer cell survival. Inhibition of the CCL2-CCR2 axis has been shown to increase the levels of cleaved caspase-3 and PARP, thereby restoring sensitivity to cabazitaxel (23). *In vivo* studies further demonstrated that combining CCL2 or CCR2 antagonists with paclitaxel or carboplatin significantly improves treatment efficacy (26). While CCL2 has been implicated in promoting EGFR-TKIs resistance, particularly via activation of the AKT pathway (27), the precise downstream signaling mechanisms underlying the effect remains largely undefined.

EMT is a complex cellular process that drives the conversion of epithelial cells into mesenchymal cells and plays a fundamental role in various physiological and pathological contexts, including embryonic development, tissue repair, and cancer metastasis (28). Increasing evidence highlights a strong association between CCL2 and the induction and regulation of EMT (20, 29). In human breast carcinoma cells, CCL2 has been shown to directly promote EMT by activating ERK-GSK3β-SNAI1 signaling pathways (30). By binding to its receptor, CCR2, CCL2 triggers downstream signaling events that lead to the loss of epithelial markers and the acquisition of mesenchymal traits, thereby enhancing cellular plasticity and invasion potential (31). EMT has also emerged as a critical mechanism contributing to resistance to EGFR-TKIs in NSCLC (32, 33). EMT-driven resistance is frequently accompanied by the activation of alternative survival pathways, including the AXL (13, 34), PI3K/AKT (35), and TGF-β signaling cascades (36), which collectively promote drug tolerance and disease progression.

In this study, we demonstrated that CCL2 confers osimertinib resistance in NSCLC, while CCL2 silencing restores osimertinib sensitivity. Moreover, our findings suggest that the CCL2–STAT3–ZEB1 axis plays a critical role in mediating this resistance. Importantly, combined inhibition of STAT3 and osimertinib effectively overcame CCL2-induced EGFR-TKI resistance. These results provide mechanistic insight into osimertinib resistance and highlight novel therapeutic targets for overcoming drug resistance in NSCLC.

## 2 Materials and methods

### 2.1 Chemicals and cell lines

Osimertinib, INCB3344 (CCR2 antagonist), and S3I201 (STAT3 inhibitor) were purchased from Selleck Chemicals (Houston, Texas). Recombinant human CCL2 protein was purchased from Peprotech (Rocky Hill, NJ). Carlumab (human anti-CCL2 antibody) was purchased from MedChemExpress (Monmouth Junction, NJ). Human lung cancer cell lines NCI-H1975 (RRID: CVCL_1511) harboring EGFR L858R and T790M mutations and HCC827 (RRID: CVCL_2063) harboring EGFR exon 19 mutation were purchased from the ATCC (Manassas, VA). The gefitinib-resistant HCC827/gef cells were selected from their parental HCC827 cells after exposure to stepwise escalating concentrations of gefitinib up to 10 μM. HCC827/gef cells were cross-resistant to osimertinib (37). The osimertinib-resistant H1975/AZD cells were developed from the parental cells subjected to persistent gradient exposure to osimertinib (AZD9291) for about 6 months by increasing osimertinib concentration up to 3 μM (13). Single clones of osimertinib-resistant H1975/AZD-15 and H1975/AZD-18 cells were obtained by isolating single cells from H1975/AZD mixed clones using a serial dilution method.

HCC827-CCL2 cells were generated by transfecting them with a plasmid carrying human CCL2 cDNA (SC118317, Origene, Rockville, MD). After transfection, these cells were selected over 14 days using geneticin-containing media. Subsequently, the cells were cultured in RPMI 1640 medium supplemented with 5% fetal bovine serum (FBS), 100 U/mL penicillin, and 100 μg/mL streptomycin (Gibco, Grand Island, NY). The cell cultures were maintained at 37 °C in a humidified incubator with 5% CO_2_. All cell lines were authenticated by short tandem repeat profiling within the last three years and confirmed to be free of mycoplasma contamination.

### 2.2 Small interfering RNA and transfection

Small interfering RNA (siRNA) specific for CCL2 (ID: s12565, ID:s12566) or ZEB1 (ID:s229971) was purchased from Life Technology. CCR2 siRNA, SMARTpool siRNAs targeting ZEB1, VIM were provided by Dharmacon (Chicago, IL). The siRNA was transfected into cells using Lipofectamine RNAiMAX Reagent (Life Technologies, CA) according to the manufacturer’s instructions.

### 2.3 Cytotoxicity assays

The cells were first plated at a density of 3000–5000 cells per well in 96-well plates. Subsequently, the cells were treated with various concentrations of drugs. After 48–72 h of treatment, cell viability was determined using 3-(4, 5-Dimethylthiazol-2-yl)-2, 5-diphenyltetrazolium bromide (MTT) assays (88417, Merck Millipore, Darmstadt, Germany) according to the manufacturer’s instructions. Finally, the absorbance was measured at 570 nm using a SpectraMax i3x Multi-Mode Microplate Reader (Molecular Device, CA). The cell survival percentages are represented as mean ± SD (SD, standard deviation).

### 2.4 Western blotting

Cells were lysed in RIPA buffer (#9806, Cell Signaling Technology, Danvers, MA, USA) supplemented with 1% phosphatase inhibitor cocktail. Equal amounts of protein (20–30 μg) were subjected to SDS–polyacrylamide gel electrophoresis (6–12% gels), followed by transfer onto polyvinylidene difluoride (PVDF) membranes (#77010, Invitrogen, NY, USA). Membranes were blocked with 5% fat-free milk in TBS for 1 hour at room temperature and then incubated with primary antibodies overnight at 4 °C. After washing, membranes were incubated with either horseradish peroxidase (HRP)-conjugated secondary antibodies or fluorescently labeled secondary antibodies for 1 hour at room temperature. All antibodies used in this study are listed in **Supplementary Table S1**. Protein bands were visualized either using immobilon Western chemiluminescent HRP Substrate (Merck) with ImageQuant LAS4000 or fluorescence detection with the Invitrogen iBright FL1500 imaging system.

### 2.5 ELISA

We used 24-hour cell-conditioned media that were controlled for cell number and media volume to detect CCL2 using human CCL2 enzyme-linked immunosorbent assay (ELISA) kits (DCP00, R&D Systems) according to the manufacturer’s instructions. Absorbance was read at an optical density of 450 nm using a SpectraMax i3x Multi-Mode Microplate Reader. The concentration of each conditioned media was determined by referring to a standard curve and is represented as the mean ± SD.

### 2.6 Cell apoptosis assay

HCC872/gef cells were transfected with CCL2 siRNA for 48 h and then exposed to 250 nM osimertinib for another 24 h. The Annexin V-FITC apoptosis detection Kit (556547, BD Bioscience) was used to detect apoptosis, following the manufacturer’s protocol. Subsequently, the cells were analyzed using an Attune™ NxT Flow Cytometer (Life Technologies).

### 2.7 Caspase-Glo 9 assay

The caspase-9 activity in cell lysates was assayed using the Caspase 9-Glo luminescent kit (G8211, Promega, Madison, WI), following the manufacturer’s instructions. Cells were seeded in 96-well plates and subsequently treated with osimertinib for 18 h. After incubation, the cells were lysed. Luminescence values were measured using the SpectraMax i3x Multi-Mode Microplate Reader equipped for luminescence detection.

### 2.8 RNA isolation and quantitative real-time PCR

Total RNA was extracted using TRIzol™ reagent (15596026, Life Technologies) according to the manufacturer’s instructions. The reverse transcription reaction was performed using 1 μg of total RNA with a Quantscript RT kit (205311, Qiagen Biotechnology, Dusseldorf, Germany). The mRNA expression level was determined by quantitative real-time PCR using SYBR Green Master Mix (4309155, Applied Biosystems, CA) and a Quantstudio 7 flex real-time PCR system (Applied Biosystems). The sequences of the gene-specific primers used in the study are listed in **Supplementary Table S2**.

### 2.9 Xenograft animal studies

All animal procedures were approved by the Institutional Animal Care and Use Committee (IACUC) of the National Taiwan University College of Medicine (IACUC Approval No: 20170159 and 20160310), Taiwan, and performed in compliance with the IACUC guidelines. Cancer cells (HCC827-mock, HCC827-CCL2, and H9175/AZD-18) were injected subcutaneously into the lower rear flank of 5-week-old severe combined immune deficient (SCID) athymic male mice from BioLASCO (Taiwan). The animals were randomized into groups 14–21 days after tumor-cell injection when tumors had grown to approximately 200 mm^3^. Tumor volumes were evaluated after treatment with osimertinib or S3I201. Tumor volume and mouse weight were recorded every 4 days, and tumor volumes were assessed using the formula mm^3^=width^2^×length/2.

### 2.10 Malignant pleural effusion isolation

From February 2012 to August 2021, pleural effusion samples were consecutively collected via thoracentesis in the chest ultrasonography examination room at National Taiwan University Hospital. Prior to thoracentesis, all enrolled patients provided written informed consent for future molecular analyses, in accordance with protocols approved by the Institutional Review Board (REC No. 201705098RINC). Only pleural effusions with cytological confirmation of lung adenocarcinoma were included in this study.

Red blood cells in the pleural fluid were first lysed using RBC lysis buffer. The remaining cells were washed twice with PBS and then cultured in complete RPMI-1640 medium (38), with media changes every 2–3 days. The cells were cultured for 10 days to reduce fibroblast contamination before harvesting. Total RNA was then extracted using TRIzol™ reagent (Invitrogen, NY), and CCL2 expression was analyzed by RT-qPCR using TaqMan CCL2 probes (Life Technologies) on the Applied Biosystems QuantStudio 7 Flex system.

### 2.11 Functional association of CCL2

Gene expression profiles of lung adenocarcinoma (LUAD) from The Cancer Genome Atlas (TCGA) were retrieved using the R package TCGAbiolinks. To assess pathway activity, single-sample enrichment scores for the Hallmark gene sets in the Molecular Signatures Database (MSigDB) were computed with the R package GSVA. The associations between CCL2 expression levels and pathway enrichment scores were evaluated using Spearman’s rank correlation coefficients. For analyses involving multiple comparisons, false discovery rate (FDR) correction was applied using standard multiple-testing adjustment procedures, and adjusted p values (q values) were used to determine statistical significance.

### 2.12 Chromatin immunoprecipitation assay

Chromatin–protein complexes were crosslinked by adding paraformaldehyde directly to the culture medium to a final concentration of 1% and quenched with 125 mM glycine for 5 min. ChIP assays were performed using the Pierce™ Magnetic ChIP Kit (26157, Thermo Scientific) according to the manufacturer’s instructions. Mouse anti-STAT3 antibody (#9139; Cell Signaling Technology) was used for immunoprecipitation, and normal mouse IgG (#68860; Cell Signaling Technology) served as the negative control. Immunoprecipitated DNA was analyzed by quantitative PCR using primers specific for the human ZEB1 promoter, as listed in **Supplementary Table 2**.

### 2.13 Statistical analysis

Data are presented as the mean ± SD for *in vitro* and *in vivo* experiments. The statistical comparison between the two groups was assessed using the unpaired Student’s *t-test*. Clinical data were presented as the group’s median, and the statistical comparison between the two groups was performed using the Mann–Whitney U test. Statistical significance was set at p < 0.05. Events were defined as tumors larger than 400 mm^3^. Survival curves of the mice were compared using the log-rank test and generated using the Kaplan-Meier method. All statistical analyses were conducted using SPSS software (version 22.0, SPSS Inc., Chicago, IL).

## 3 Results

### 3.1 The chemokines S100A8 and CCL2 emerged as high-ranking genes associated with TKI resistance in public gene expression datasets

Previously, to identify genes associated with EGFR-TKI resistance, we analyzed four gene expression datasets (GSE80344, GSE106765, GSE103350, and GSE95558). Gene scores were calculated based on expression levels, and a weighted ranking system was employed to integrate the results across datasets. The methodologies for data integration, TKI-associated gene prioritization, and candidate gene alteration analysis have been described in detail in previous studies (39). Among the selected candidate genes, IGFBP7 ranked highest, with a resistance score of 3.85. We previously demonstrated that IGFBP7 contributes to the mechanisms of EGFR-TKI resistance and represents a potential therapeutic target (40). In the present study, we further explored novel cytokine signaling pathways involved in EGFR-TKI resistance. Among the top 20 upregulated genes identified (**Supplementary Table S3**), the EGFR-TKI resistance candidate genes S100A8 and CCL2 were selected. Both chemokines are associated with immune modulation, tumor microenvironment, and tumor progression. These genes exhibited resistance scores of 3.45 and 2.30, respectively. In this study, we will investigate the role of CCL2 in mediating resistance to EGFR-TKIs in NSCLC. Although CCL2 has been implicated in promoting such resistance, the precise downstream mechanisms that confer EGFR-TKI resistance remain incompletely defined. Further research is needed to clarify these signaling pathways and to develop effective strategies for overcome the CCL2-induced EGFR-TKI resistance.

### 3.2 CCL2 expression is significantly increased in malignant pleural effusions and osimertinib-resistant lung cancer cells

**Figure 1:**
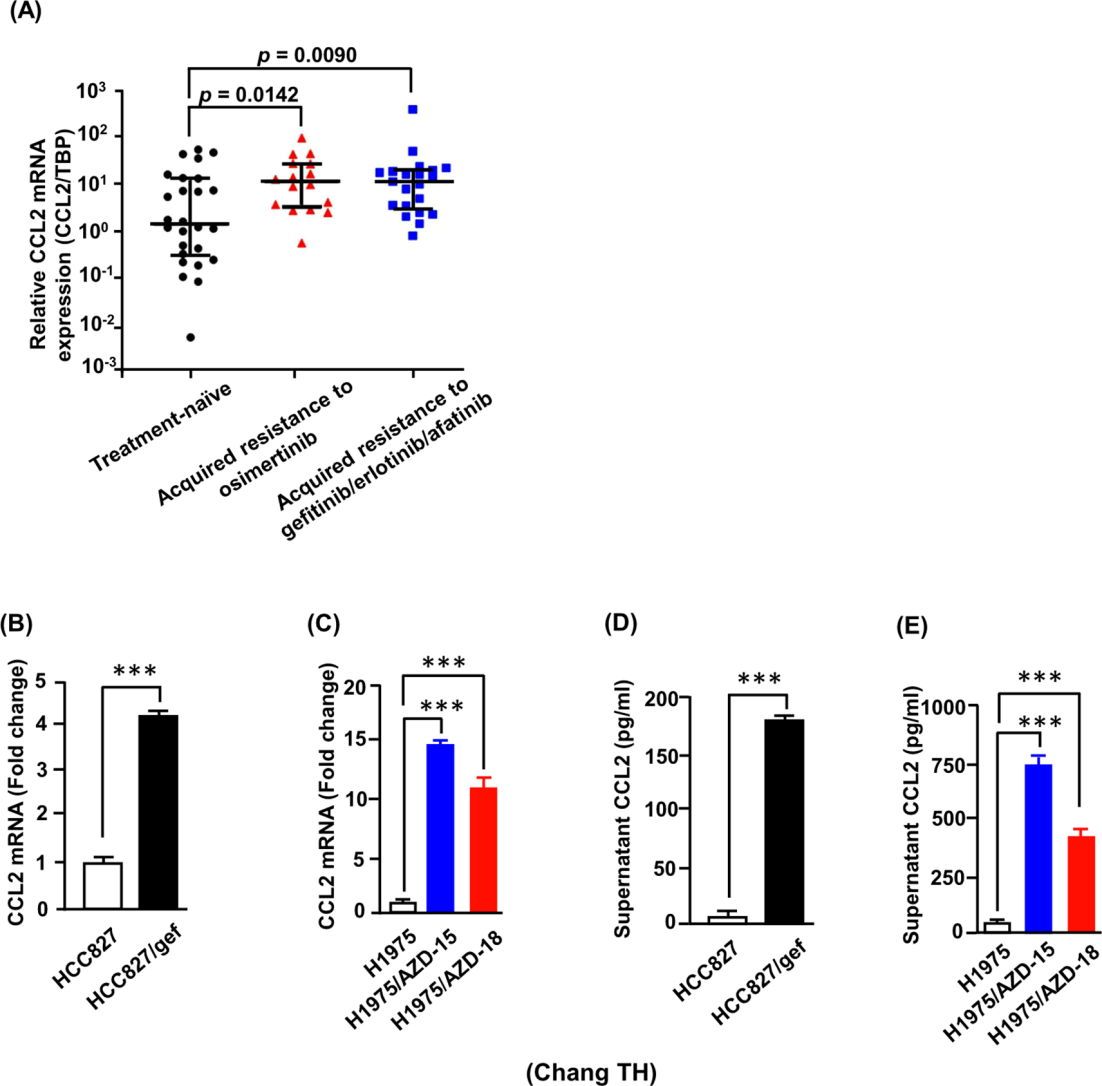
CCL2 expression is elevated in malignant pleural effusions and osimertinib-resistant cells. **(A)** Relative CCL2 mRNA expression in primary tumor cells isolated from malignant pleural effusions (MPEs) of lung adenocarcinoma patients harboring EGFR mutations. Samples were obtained from treatment-naïve patients (n = 26), patients with acquired resistance to osimertinib (n = 16), and patients with resistance to first- or second-generation EGFR-TKIs (gefitinib, erlotinib, or afatinib; n = 21). Each data point represents an independent biological sample. Data are presented as median with interquartile range. Statistical comparisons were performed using the Mann–Whitney U test; effect size (rank-biserial correlation) is shown where applicable. **(B, C)** Quantitative RT-PCR analysis of CCL2 mRNA expression in parental and resistant cell lines. Data represent the mean ± SD from three independent biological experiments, each performed in triplicate (***p < 0.001, Student’s *t-test*). **(D, E)** Secreted CCL2 protein levels measured by ELISA in the conditioned media of the same cell lines. Data represent the mean ± SD from three independent biological experiments (***p < 0.001, Student’s *t-test*).

To validate whether the candidate gene CCL2 is involved in acquired resistance to EGFR-TKIs, we analyzed a total of 63 malignant pleural effusion (MPE) samples collected from patients with lung adenocarcinoma harboring EGFR mutations. Of these, 26 samples were obtained at the time of initial diagnosis prior to systemic treatment, 16 were collected after the development of resistance to osimertinib, and 21 were derived from patients with acquired resistance to first- or second-generation EGFR-TKIs (erlotinib: 10, gefitinib: 10, afatinib: 1). No significant differences in clinical characteristics were observed among the three patient groups (**Supplementary Table S4**). CCL2 expression levels in primary cancer cells isolated from MPEs were quantified using RT-qPCR. The results showed that median CCL2 expression was significantly elevated in MPEs from patients with osimertinib resistance compared to treatment-naïve samples (p = 0.0142, Mann–Whitney U test; **Figure 1A**). Similarly, patients with resistance to first- or second-generation EGFR-TKIs also exhibited significantly higher median CCL2 expression (p = 0.0090, Mann–Whitney U test; **Figure 1A**). These findings suggest that CCL2 is upregulated in malignant pleural effusions from lung adenocarcinoma patients following resistance to osimertinib and first-/second-generation EGFR-TKIs.

To further explore the potential role of CCL2 in mediating osimertinib resistance, we assessed CCL2 expression in a panel of established EGFR-TKI-resistant cell lines. Quantitative RT-PCR revealed significantly increased CCL2 mRNA levels in HCC827/gef, H1975/AZD-15, and H1975/AZD-18 cells compared with their respective parental lines (**Figures 1B, C**). Consistently, ELISA assays confirmed markedly elevated CCL2 protein secretion in the resistant cells (**Figures 1D, E**), which was further supported by results from a human cytokine array (**Supplementary Figure S1**). Notably, the cytokine array also identified interleukin-8 (IL8) as another differentially expressed cytokine. Our previous research demonstrated that IL8 contributes to EGFR-TKI resistance (41). Collectively, these results indicate that CCL2 is consistently upregulated in malignant pleural effusions and in osimertinib-resistant lung cancer cells, implicating it as a potential mediator of acquired resistance.

### 3.3 CCL2 regulates osimertinib sensitivity by modulating apoptosis in lung cancer cells

**Figure 2:**
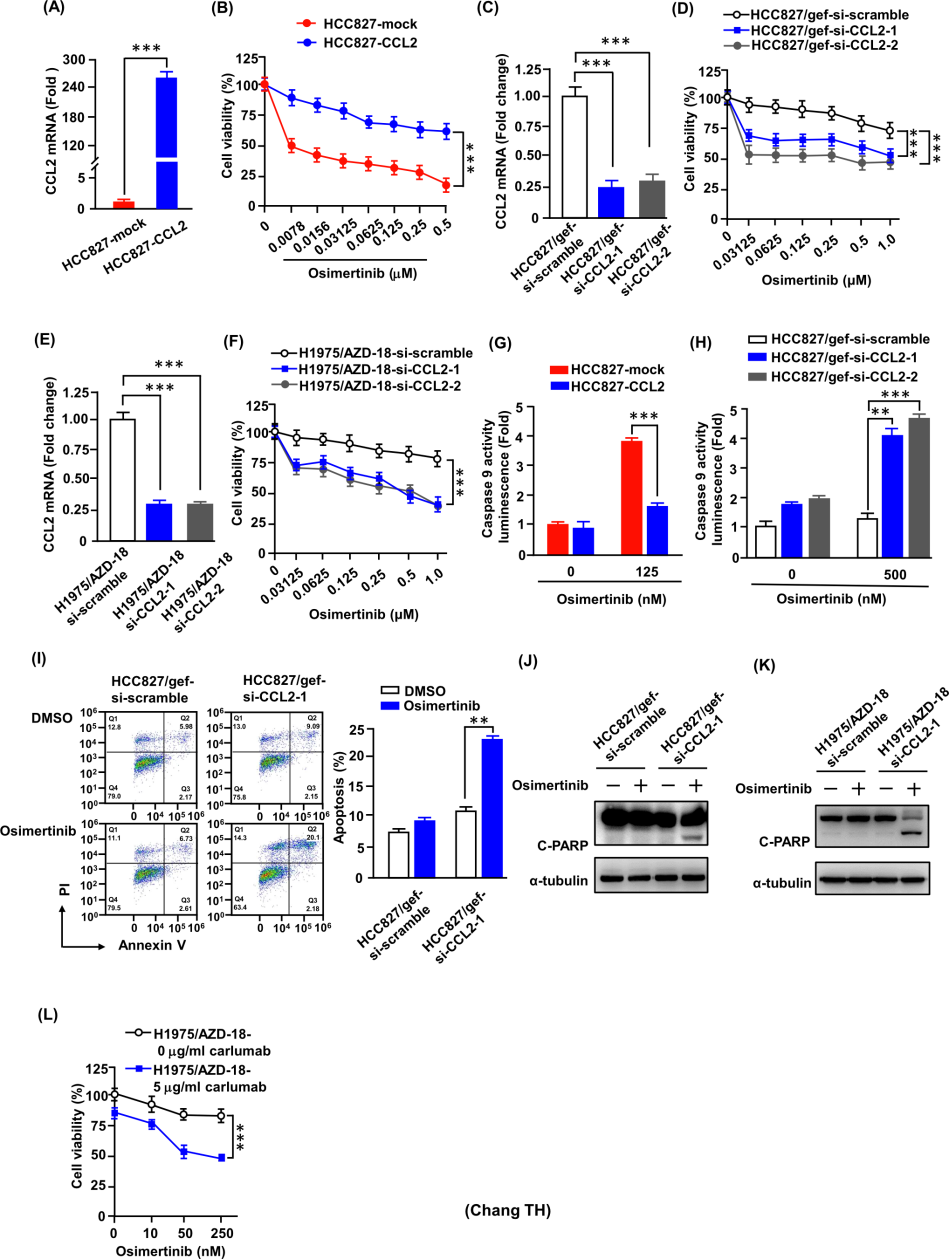
Gain- and loss-of-function analyses reveal that CCL2 regulates osimertinib resistance by suppressing apoptosis. **(A)** CCL2 mRNA levels in HCC827-mock and HCC827-CCL2 cells were quantified by RT-qPCR (***p < 0.001). **(B)** Cell viability after 48 h of osimertinib treatment was assessed by MTT assay. DMSO-treated cells were normalized to 100%. Data represent the mean ± SD from four independent experiments, each performed in triplicate (***p < 0.001, Student’s t-test). **(C)** CCL2 mRNA levels in HCC827/gef cells transfected with CCL2 siRNA were quantified by RT-qPCR. Data represent the mean ± SD from three independent biological replicates (***p < 0.001, Student’s t-test). **(D)** Cell viability after 72 h of osimertinib treatment was measured by MTT assay. **(E)** CCL2 mRNA levels in H1975/AZD-18 cells transfected with CCL2 siRNA were quantified by RT-qPCR. Data represent the mean ± SD from three independent biological replicates (***p < 0.001, Student’s t-test). **(F)** Osimertinib sensitivity in si-CCL2 cells was determined by MTT assay. Data represent the mean ± SD from three independent biological replicates (***p < 0.001, Student’s t-test). **(G)** Caspase-9 activity in HCC827-CCL2 cells after osimertinib treatment (***p < 0.001). **(H)** Caspase-9 activity in CCL2-knockdown cells following 500 nM osimertinib exposure for 24 h (**p < 0.01; ***p < 0.001, Student’s t-test). **(I)** Flow cytometry analysis of apoptosis (Annexin V/PI) in HCC827/gef cells. The bar chart displays the percentage of apoptotic cells derived from three independent experiments (**p < 0.01 compared with vehicle control, Student’s *t-test*). **(J, K)** Western blot analysis of cleaved PARP and α-tubulin in CCL2-knockdown cells treated with osimertinib. Representative blots are shown from three independent experiments. **(L)** Cell viability of H1975/AZD-18 cells treated with osimertinib in the presence or absence of the CCL2-neutralizing antibody carlumab was assessed by MTT assay. Data represent the mean ± SD from three independent experiments (***p < 0.001, Student’s t-test).

To validate the functional significance of CCL2 in mediating resistance to osimertinib, we generated CCL2-overexpressing transfectants (HCC827-CCL2) and corresponding control transfectants (HCC827-mock; **Figure 2A**). Introduction of CCL2 into osimertinib-sensitive HCC827 cells conferred resistance to osimertinib, as demonstrated by MTT assay (**Figure 2B**).

As shown in **Figures 2C, D**, transient transfection of CCL2 siRNA into osimertinib-resistant cells (HCC827/gef and H1975/AZD-18) effectively reduced CCL2 expression compared with their respective controls transfected with scramble siRNA (HCC827/gef-si-scramble and H1975/AZD-18-si-scramble). Notably, CCL2 knockdown in these resistant cells led to a significant increase in cell death upon osimertinib treatment (**Figures 2E, F**).

To further validate these findings, stable CCL2-knockdown clones were generated using CCL2 shRNA in the same resistant cells, achieving inhibition efficiencies of 93%, 88%, and 90% in HCC827/gef, H1975/AZD-15, and H1975/AZD-18, respectively (**Supplementary Figures S2A, C, E**). These stable knockdown clones exhibited an even greater extent of osimertinib-induced cell death, reinforcing the results observed with transient siRNA transfection (**Supplementary Figures S2B, D, F**).

We next assessed the impact of CCL2 on osimertinib-induced apoptosis. In sensitive HCC827 cells, CCL2 overexpression significantly reduced caspase-9 activity triggered by osimertinib treatment (**Figure 2G**). Conversely, in HCC827/gef cells, CCL2 downregulation markedly enhanced osimertinib-induced apoptosis, as confirmed by luminescent caspase-9 activity assay and Annexin V-FITC apoptosis staining (**Figures 2H, I**). Furthermore, osimertinib-induced PARP cleavage was significantly increased in HCC827/gef and H1975/AZD-18 cells following CCL2 knockdown by siRNA transfection (**Figures 2J, K**). Importantly, pharmacological neutralization of extracellular CCL2 using the CCL2-neutralizing antibody carlumab significantly enhanced osimertinib-induced cytotoxicity in H1975/AZD-18 cells, as assessed by MTT assay (**Figure 2L**). Collectively, these findings underscore the critical role of CCL2 in sustaining osimertinib resistance in lung cancer cells through the modulation of apoptotic responses.

### 3.4 CCL2 overexpression attenuates the antitumor efficacy of osimertinib *in vivo*

**Figure 3:**
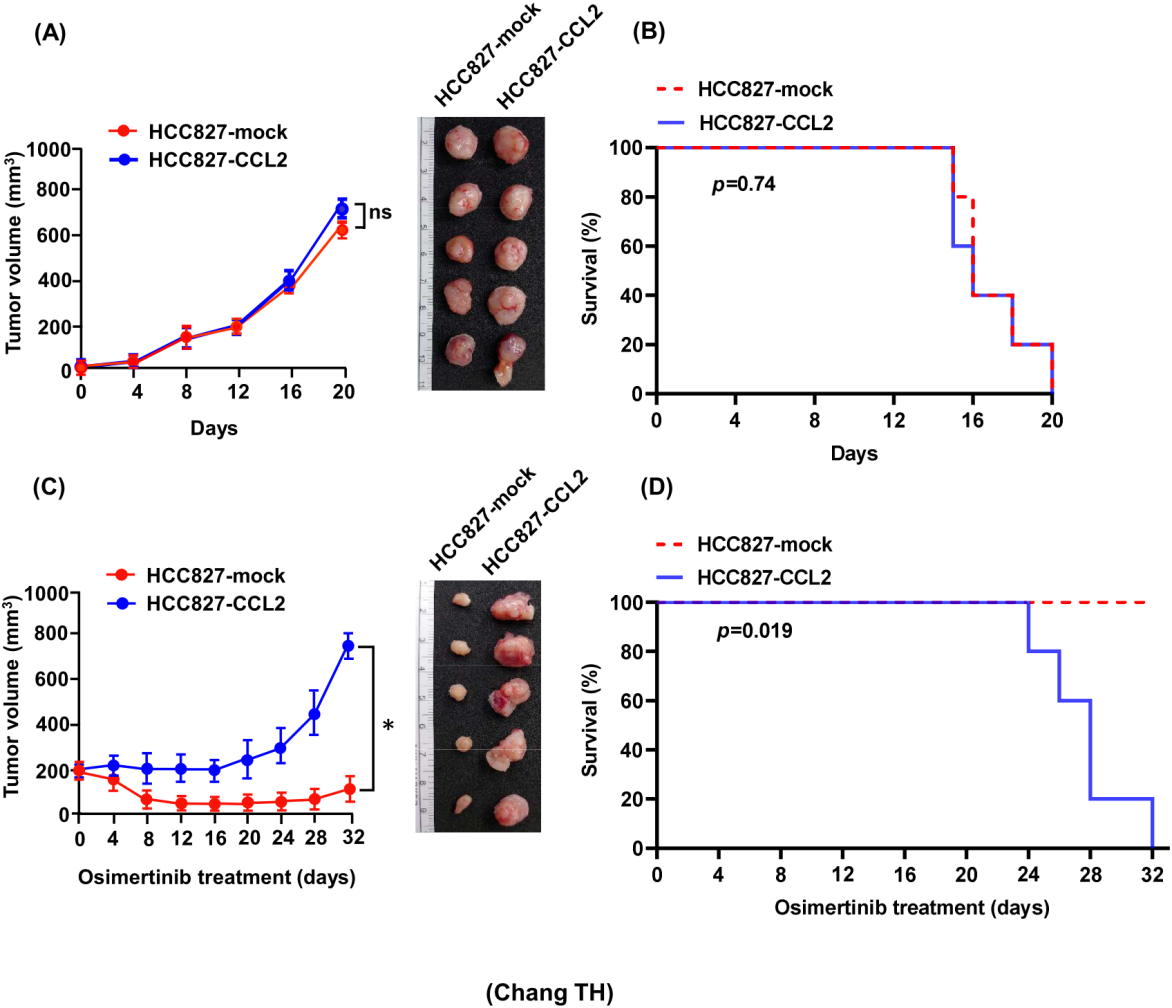
CCL2 overexpression attenuates the antitumor efficacy of osimertinib and shortens survival in xenograft models. **(A)** Growth curves of HCC827-CCL2 and HCC827-mock xenografts in SCID mice (n = 6 per group). Tumor volumes were measured every 4 days and expressed as mean ± standard error (SE). ns, not significant. **(B)** Kaplan–Meier survival curves comparing mice bearing HCC827-CCL2 and mock xenografts. No significant difference was observed between groups (log-rank test, p > 0.05). **(C)** Tumor growth of mice treated with osimertinib (0.5 mg/kg/day) for 32 days (n = 6 per group). Data are presented as mean ± SE (*p < 0.05, Student’s *t-test*). **(D)** Kaplan–Meier survival analysis of osimertinib-treated xenograft-bearing mice (HCC827-CCL2 vs mock). Survival was defined by tumor volume <400 mm³. Statistical significance was determined by the log-rank (Mantel–Cox) test (p = 0.019).

*In vivo* experiments were performed to assess the effects of CCL2 on tumor growth both in the absence and presence of osimertinib. There was no significant difference in tumor growth or survival between mice injected with HCC827-mock cells and those injected with HCC827-CCL2 cells in the absence of treatment (**Figures 3A, B**). Notably, osimertinib administration markedly inhibited tumor growth in mice bearing HCC827-mock tumors, but this inhibitory effect was substantially attenuated in mice bearing HCC827-CCL2 tumors (**Figure 3C**). In xenograft survival studies, survival was defined as maintaining tumor volume below 400 mm³. A significant difference in survival duration was observed between osimertinib-treated mice bearing HCC827-mock tumors and those bearing HCC827-CCL2 tumors (**Figure 3D**). Collectively, these findings demonstrate that elevated CCL2 expression diminishes the antitumor efficacy of osimertinib in lung cancer cells.

### 3.5 The STAT3 and ERK signaling pathways contribute to CCL2-mediated osimertinib resistance

**Figure 4:**
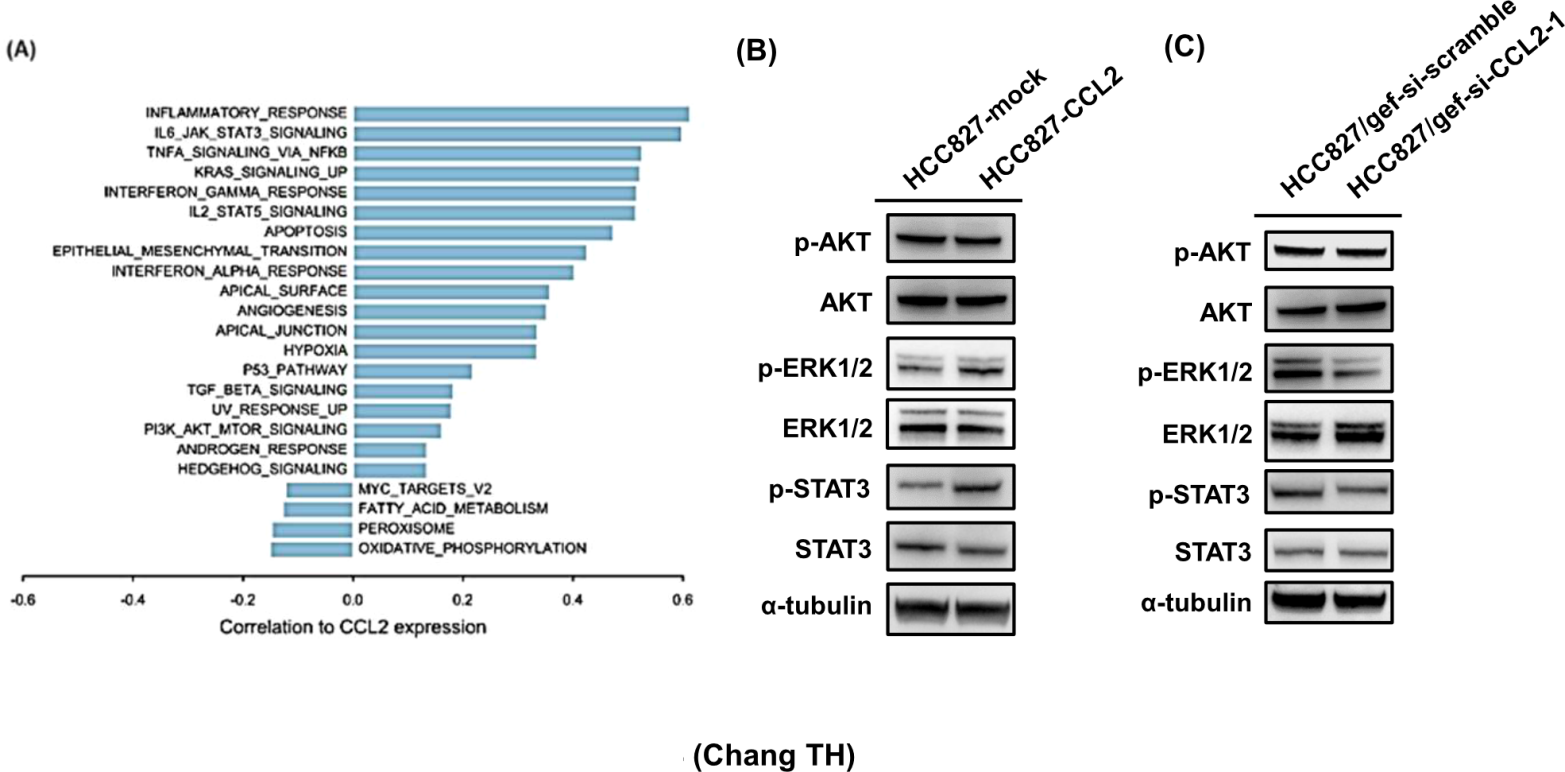
CCL2 activates STAT3 and ERK. **(A)** Spearman’s correlation coefficients were calculated between CCL2 expression and the enrichment scores of MSigDB Hallmark gene sets derived from TCGA-LUAD transcriptomes. Only significantly correlated pathways (FDR < 0.01) are shown. **(B)** Western blot analysis of phosphorylated STAT3 and ERK1/2 in HCC827-CCL2 cells compared with vector control. CCL2 overexpression led to increased phosphorylation of STAT3 and ERK1/2, while p-AKT levels remained unchanged. **(C)** Phosphorylated STAT3 and ERK1/2 in HCC827/gef-si-CCL2 cells compared with control transfectants. Knockdown of CCL2 reduced p-STAT3 and p-ERK1/2 levels. α-tubulin served as the loading control. Representative blots are shown from three independent biological experiments.

To further explore the clinical and biological relevance of CCL2 expression in lung adenocarcinoma, we analyzed TCGA-LUAD transcriptomic data. CCL2 expression was positively correlated with multiple inflammation- and cytokine-related pathways, including inflammatory response, IL6/JAK/STAT3 signaling, and TNFα signaling via NF-κB. Additional associations were observed with KRAS, interferon-γ, and epithelial–mesenchymal transition (EMT) pathways (FDR < 0.01; **Figure 4A**). In contrast, metabolic pathways such as oxidative phosphorylation and fatty acid metabolism exhibited weak or negative correlations with CCL2 expression. Furthermore, Kaplan–Meier survival analysis revealed that high CCL2 expression was significantly associated with shorter overall survival in lung cancer patients (p < 0.05; **Supplementary Figure S3**). Collectively, these results support the association of CCL2 with IL6/JAK/STAT3 signaling, EMT activation, pro-inflammatory signaling, and poor clinical outcomes in NSCLC.

Since ERK is another key downstream effector of EGFR signaling and may interact with STAT3 activity, we further examined whether CCL2 modulates STAT3 and ERK signaling. CCL2-overexpressing HCC827 cells exhibited elevated basal levels of phosphorylated STAT3 and ERK1/2, with no change in basal AKT phosphorylation (**Figure 4B**). In contrast, CCL2 knockdown in HCC827/gef cells resulted in a marked reduction in STAT3 and ERK1/2 phosphorylation (**Figure 4C**). These findings suggest that CCL2 selectively activates STAT3 and ERK signaling, but not the AKT pathway, in EGFR-mutant lung cancer cells.

### 3.6 Osimertinib-resistant cells exhibit EMT features and activation of ERK and STAT3

**Figure 5:**
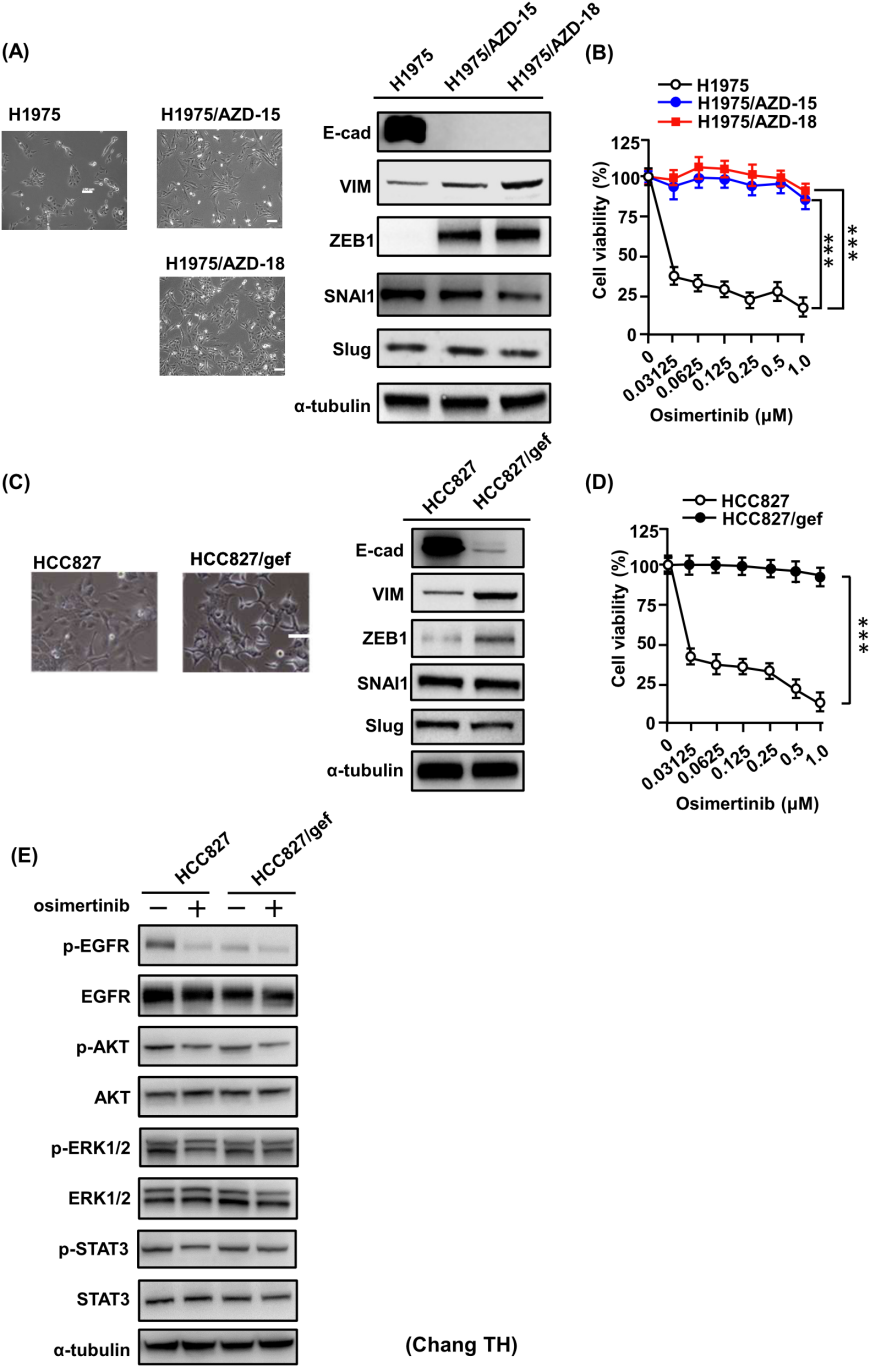
Osimertinib-resistant cells display EMT features and sustained activation of ERK and STAT3 signaling. **(A)** Phase-contrast images of H1975 (osimertinib-sensitive), H1975/AZD-15, and H1975/AZD-18 (resistant) cells. H1975/AZD-15 and H1975/AZD-18 cells exhibited elongated, spindle-shaped morphology indicative of EMT. Scale bar, 100 μm. Western blot analysis of EMT markers (E-cadherin, vimentin) and transcription factors (ZEB1, SNAI1, Slug) is shown on the right. **(B)** Dose–response curves for osimertinib in parental (H1975) and resistant (H1975/AZD-15 and H1975/AZD-18) NSCLC cells. Cell viability was measured by MTT assay. Data represent the mean ± SD from four independent biological experiments, each performed in triplicate (***p < 0.001, Student’s *t*-test). **(C)** Phase-contrast images of HCC827 (sensitive) and HCC827/gef (resistant) cells. EMT marker expression was evaluated by Western blot, revealing increased ZEB1 expression in resistant cells. **(D)** Dose–response curves for osimertinib in parental (HCC827) and resistant (HCC827/gef) NSCLC cells. Cell viability was measured by MTT assay. Data represent the mean ± SD from four independent biological experiments, each performed in triplicate (***p < 0.001, Student’s *t*-test). **(E)** Western blot analysis of EGFR and downstream signaling molecules (p-AKT, p-ERK1/2, and p-STAT3) in HCC827 and HCC827/gef cells treated with or without osimertinib (250 nM, 3 h). Representative blots are shown from three independent biological experiments.

Previous studies have established a link between EMT and both primary and acquired resistance to EGFR-TKIs (33, 42–44). Morphologically, osimertinib-resistant cells (H1975/AZD-15 and H1975/AZD-18) displayed distinct EMT-like features compared with their osimertinib-sensitive parental counterparts (**Figure 5A**). In our models, resistant cells exhibited a pronounced reduction in the epithelial marker E-cadherin and increased expression of the mesenchymal markers Vimentin and ZEB1. In contrast, the expression of other EMT-associated transcription factors, such as SNAI1 and SLUG, remained unchanged between sensitive and resistant cells. These resistant clones were established through stepwise escalation of osimertinib concentrations up to 3 μM, as described previously (13). H1975/AZD-18 cells maintained robust proliferation despite osimertinib treatment, confirming their resistant phenotype, whereas parental H1975 cells showed marked growth inhibition at concentrations below 30 nM (**Figure 5B**). Similar phenotypic changes were observed in gefitinib-resistant HCC827/gef cells (**Figure 5C**), which were derived through gradual dose escalation up to 10 μM gefitinib (40). HCC827/gef cells retained strong proliferative capacity under osimertinib exposure, whereas parental HCC827 cells exhibited marked sensitivity (**Figure 5D**). To further explore downstream signaling, we examined the phosphorylation status of EGFR and its key downstream effector following osimertinib treatment. In sensitive HCC827 cells, osimertinib effectively suppressed phosphorylation of EGFR, AKT, STAT3, and ERK1/2. By contrast, in resistant HCC827/gef cells, osimertinib inhibited EGFR and AKT phosphorylation but failed to fully suppress STAT3 and ERK1/2 phosphorylation (**Figure 5E**). Collectively, these findings indicate that osimertinib-resistant cells undergo EMT and maintain sustained STAT3 and ERK1/2 activation, which may contribute to drug resistance through EGFR-independent mechanisms.

### 3.7 ZEB1 knockdown restores sensitivity to osimertinib in resistant cells

**Figure 6:**
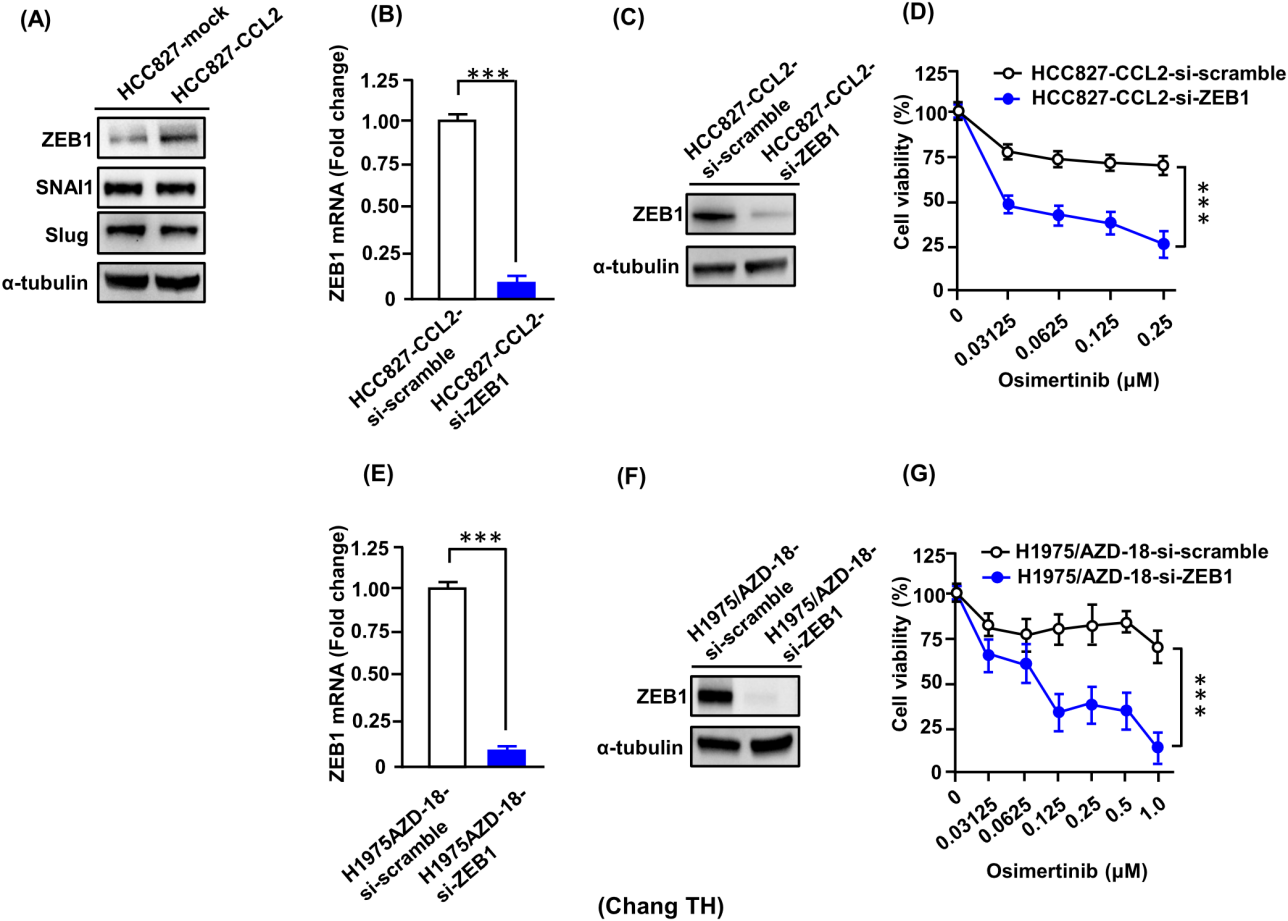
Targeting ZEB1 reverses osimertinib resistance in lung cancer cells. **(A)** Western blot analysis of EMT-related transcription factors in HCC827-CCL2 overexpression cells compared with vector control. ZEB1 expression levels increased upon CCL2 overexpression. Representative blots are shown from three independent experiments. **(B)** RT-qPCR and **(C)** Western blot analysis confirming ZEB1 knockdown efficiency in HCC827-CCL2 cells. Data represent the mean ± SD from three independent biological experiments (***p < 0.001, Student’s *t*-test). **(D)** MTT assay showing that ZEB1 knockdown restored osimertinib sensitivity in HCC827-CCL2 cells. Data represent the mean ± SD from four independent biological experiments, each performed in triplicate (***p < 0.001, Student’s *t*-test). **(E)** RT-qPCR and **(F)** Western blot analysis confirming ZEB1 knockdown efficiency in H1975/AZD-18 cells. Data represent the mean ± SD from three independent biological experiments (***p < 0.001, Student’s *t*-test). **(G)** MTT assay showing that ZEB1 knockdown restored osimertinib sensitivity in H1975/AZD-18 cells. Data represent the mean ± SD from four independent biological experiments, each performed in triplicate (***p < 0.001, Student’s *t*-test).

CCL2 has previously been implicated in promoting EMT in various cancer types, including head and neck squamous cell carcinomas (45), breast carcinoma cells (30), and glioblastoma (46). To examine whether CCL2 modulates EMT in drug-sensitive lung cancer cells, we analyzed the expression of key EMT transcription regulators in CCL2-overexpressing transfectants (HCC827-CCL2). These cells exhibited elevated ZEB1 protein levels (**Figure 6A**), while the expression levels of SNAI1 and Slug remained unchanged compared to control cells. Conversely, CCL2 knockdown via shRNA in HCC827-CCL2 and H1975/AZD-18 cells led to reduced ZEB1 expression without altering SNAI1 or Slug levels (**Supplementary Figure S4A**). To provide phenotypic evidence of EMT, immunofluorescence staining was performed for EMT markers. Compared with mock-transfected cells, HCC827-CCL2 cells showed increased vimentin staining and reduced membranous E-cadherin staining intensity (**Supplementary Figure S4B**). Consistent with these EMT-associated changes, CCL2 enhanced the migratory capacity of HCC827 cells in a transwell assay (**Supplementary Figure S5A**). In addition, recombinant human CCL2 (rCCL2) promoted spheroid formation in H1975 cells, increasing both spheroid number and size (**Supplementary Figure S5B**). These findings indicate that CCL2 not only induces ZEB1 expression but also promotes the motility and stem-like properties of lung cancer cells.

To further elucidate the role of ZEB1 in CCL2-mediated osimertinib resistance, we examined the effects of ZEB1 knockdown in CCL2-overexpressing cells. Silence ZEB1 in HCC827-CCL2 transfectants (HCC827-CCL2-si-ZEB1) markedly suppressed CCL2-induced resistance to Osimertinib (**Figures 6B–D**). Additionally, stable ZEB1 knockdown clones generated using ZEB1 shRNA in HCC827-CCL2 cells achieving approximately 80% knockdown efficiency (**Supplementary Figure S6A**), and these clones exhibited enhanced osimertinib-induced cell death, consistent with the transient knockdown results (**Supplementary Figure S6B**).

To compare the roles of ZEB1 and VIM in osimertinib-resistant HCC827-CCL2 cells, we co-transfected HCC827 cells with pooled siRNA targeting ZEB1 and VIM (**Supplementary Figure S7A**). Knockdown of either ZEB1 or VIM increased osimertinib sensitivity. Notably, dual knockdown of ZEB1 and VIM further enhanced cell death, surpassing that of VIM knockdown alone but not ZEB-1 alone (**Supplementary Figure S7B**). These results underscore the pivotal role of ZEB1 in mediating osimertinib resistance induced by CCL2.

Furthermore, ZEB1 knockdown enhanced the sensitivity of H1975/AZD-18 cells to osimertinib, as evidenced by a significant decrease in the IC_50_ values (**Figures 6E–G**). These findings support the conclusion that CCL2 contributes to osimertinib resistance in lung cancer cells through the upregulation of ZEB1 expression.

### 3.8 Targeting the CCL2-CCR2 signaling pathway enhances osimertinib-induced cell death and decreases ZEB1

**Figure 7:**
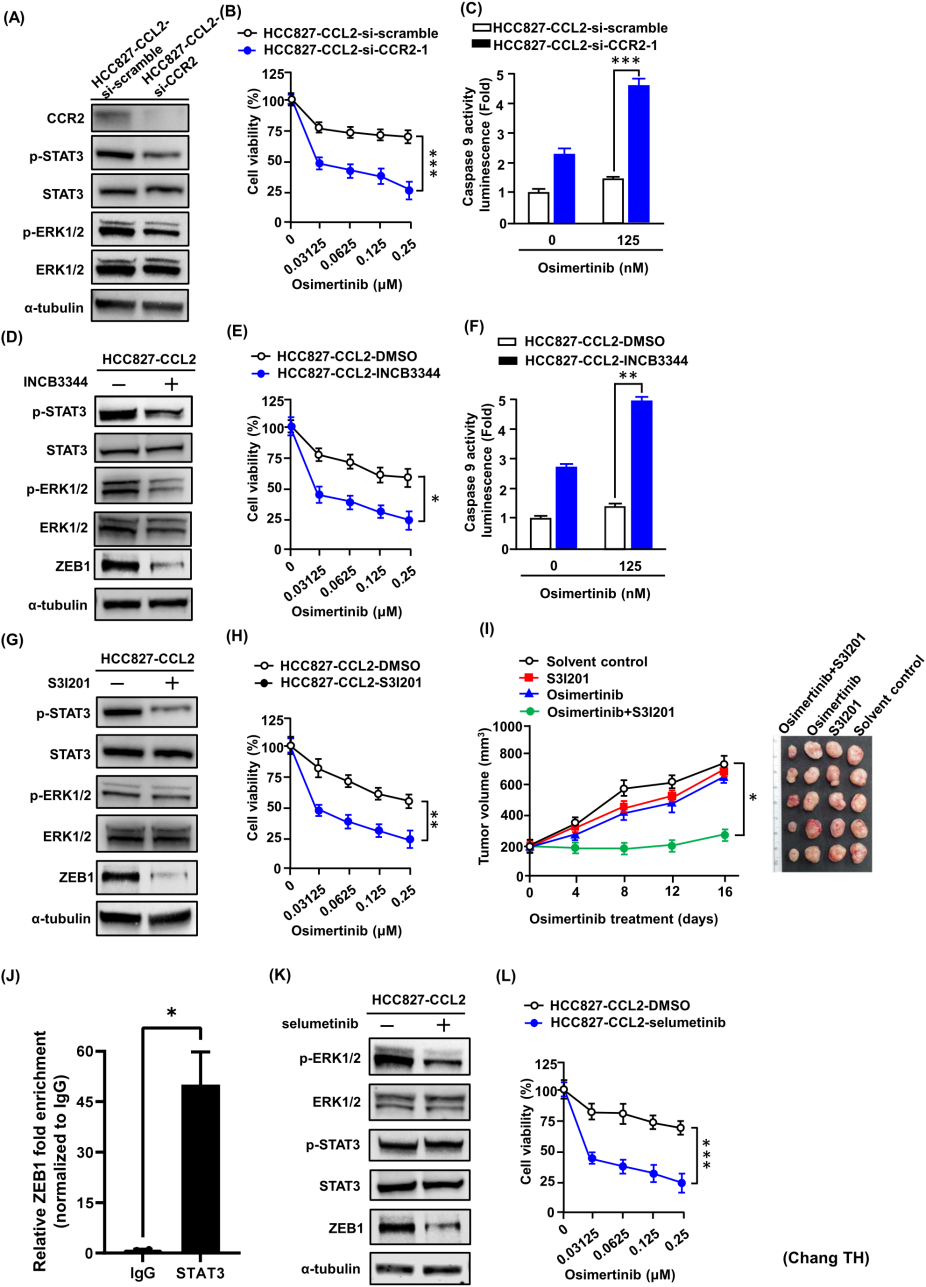
Blocking CCR2–STAT3/ERK signaling restores osimertinib sensitivity **(A)** Western blot analysis of CCR2 expression in HCC827-CCL2 cells after transfection with CCR2 siRNA. Representative blots are shown from three independent biological experiments. **(B)** MTT assay showing reduced viability of HCC827-CCL2-si-CCR2 cells treated with osimertinib (48 h). Data represent the mean ± SD from three independent biological experiments, each performed in triplicate (***p < 0.001, Student’s *t-test*). **(C)** Caspase-9 activity increased in CCR2-knockdown cells upon osimertinib treatment, measured by luminescent Caspase-Glo 9 assay. Data represent the mean ± SD from three independent experiments (***p < 0.001 vs. si-scramble, Student’s *t-test*). **(D)** Western blot analysis of downstream signaling proteins (p-STAT3, p-ERK1/2, ZEB1) in HCC827-CCL2 cells treated with CCR2 antagonist INCB3344. Representative blots are shown from three independent experiments. **(E)** Combined treatment with osimertinib and INCB3344 decreased cell viability in HCC827-CCL2 cells (MTT assay). Data represent the mean ± SD from three independent biological replicates (*p < 0.05, Student’s *t-test*). **(F)** INCB3344 enhanced caspase-9 activity upon osimertinib treatment. Data represent the mean ± SD from three independent biological experiments (**p < 0.01, Student’s *t-test*). **(G)** HCC827-CCL2 cells treated with STAT3 inhibitor (S3I201, 40 μM, 24 h) were analyzed by Western blot for p-STAT3, p-ERK1/2, and ZEB1. Representative blots are shown from three independent experiments. **(H)** MTT assay showing that STAT3 inhibition sensitized HCC827-CCL2 cells to osimertinib treatment. Data represent the mean ± SD from three independent experiments (**p < 0.01, Student’s *t-test*). **(I)** Tumor volume of H1975/AZD-18 xenografts treated with vehicle, osimertinib (1mg/kg/day), S3I201 (5mg/kg/2day), or a combination of both drugs for 16days (n = 5 in each subgroup). Data are presented as mean ± SE (*p < 0.05, Student’s *t-test)*. **(J)** Chromatin immunoprecipitation (ChIP) assay demonstrating STAT3 binding to the ZEB1 promoter in H1975/AZD-18 cells. Enrichment of ZEB1 promoter fragments was quantified by qPCR and normalized to IgG control. Data represent the mean ± SD from three independent experiments (*p < 0.05, Student’s t-test). **(K)** The expression of p-STAT3, p-ERK1/2, STAT3, ERK, and ZEB1 in HCC827-CCL2 cells with ERK inhibitor selumetinib was measured using Western blot analysis. Representative blots are shown from three independent experiments. **(L)** HCC872-CCL2 cells were treated with osimertinib and selumetinib treatment alone or in combination for 48 h; cell viability was determined using MTT assays. Data represent the mean ± SD from three independent experiments (**p < 0.001, Student’s *t-test*).

To elucidate whether CCL2 contributing to the development or maintenance of osimertinib resistance through activation STAT3 and ERK signaling pathways, we performed both genetic and pharmacological inhibition studies in CCL2-overexpressing cells. Knockdown of the CCL2 receptor CCR2 not only reduced its expression but also decreased the phosphorylation levels of STAT3 and ERK1/2 (**Figure 7A**). This genetic silencing of CCR2 significantly enhanced osimertinib-induced cytotoxicity and caspase-9 activity in HCC827-CCL2 cells (**Figures 7B, C**). Consistently, pharmacological inhibition of CCR2 using INCB3344 reduced downstream signaling activity, including phosphorylation of STAT3, ERK1/2, and the EMT regulator ZEB1, as shown by Western blotting (**Figure 7D**). Co-treatment with INCB3344 and osimertinib significantly decreased cell viability and increased caspase-9 activity (**Figures 7E, F**), and exerted a synergistic effect (**Supplementary Figure S8A**). In parallel, inhibition of STAT3 using S3I-201 suppressed CCL2-induced ZEB1 expression (**Figure 7G**) and restored sensitivity to osimertinib *in vitro* (**Figure 7H**), with synergistic effects observed upon combined treatment (**Supplementary Figure S8B**). Importantly, chromatin immunoprecipitation (ChIP) analysis demonstrated direct binding of STAT3 to the ZEB1 promoter in H1975/AZD-18 cells, providing mechanistic evidence that STAT3 transcriptionally regulates ZEB1 expression in osimertinib-resistant cells (**Figure 7J**). To further validate these findings *in vivo*, xenograft tumors were generated by injecting H1975/AZD-18 cells into SCID mice. While osimertinib monotherapy failed to suppress tumor growth, its combination with S3I-201 significantly inhibited tumor progression (**Figure 7I**). Additionally, treatment with selumetinib, an ERK inhibitor, suppressed CCL2-induced ZEB1 expression (**Figure 7K**) and reversed osimertinib resistance in HCC827-CCL2 cells (**Figure 7L**). Together, these results demonstrated that the CCL2–CCR2 axis contributes to osimertinib resistance in EGFR-TKI sensitive cells by activating the STAT3 and ERK signaling pathways.

## 4 Discussion

**Figure 8:**
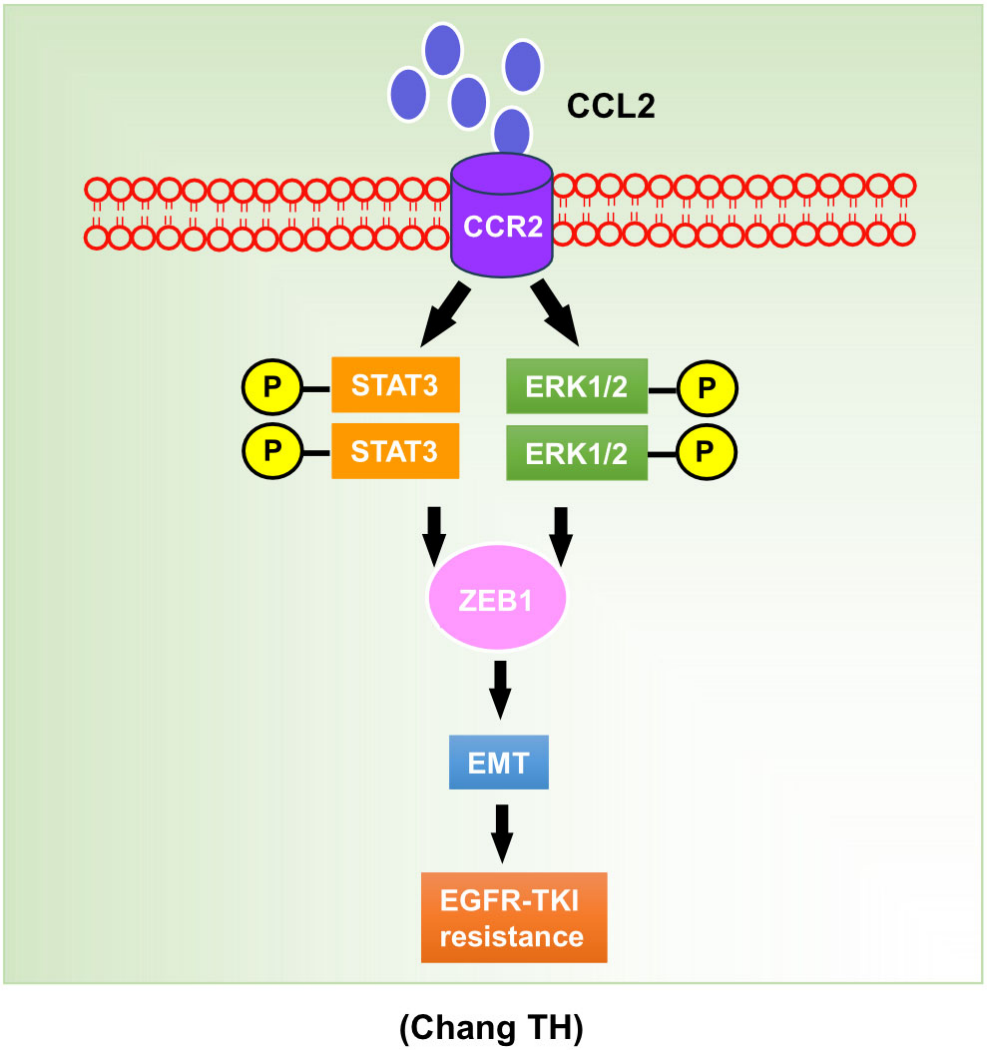
Model of the regulatory signaling networks of CCL2-CCR2-STAT3/ERK-ZEB1 in osimertinib resistance.

In this study, we investigated the role of CCL2 in the development of resistance to osimertinib in lung cancer. Higher levels of CCL2 expression were present in osimertinib-resistant cell lines, and in clinical samples following the development of osimertinib resistance. Genetic silencing of CCL2 in resistant cells significantly increased their sensitivity to osimertinib, suggesting that CCL2 plays a functional role in mediating resistance. To elucidate the underlying molecular mechanisms, we examined the downstream signaling pathways activated by CCL2. Our findings revealed that CCL2 upregulates ZEB1 expression by activating the STAT3 and ERK pathways. Pharmacological inhibition of CCR2, STAT3, and ERK signaling in CCL2-overexpressing cells significantly enhanced osimertinib-induced cell death. In summary, this is the first study to demonstrate that the CCL2–CCR2–STAT3/ERK–ZEB1 axis mediates acquired resistance to osimertinib in lung cancer. Targeting the CCL2–CCR2 signaling pathway may enhance the therapeutic efficacy of osimertinib and offer a promising strategy to overcome drug resistance (**Figure 8**).

Although EMT is a multifaceted process involving molecular, morphological, and functional alterations, our study primarily focused on the molecular and functional aspects. In addition, we examined phenotypic changes associated with epithelial–mesenchymal transition. Immunofluorescence staining revealed that modulation of CCL2 altered the cellular distribution of canonical EMT markers, characterized by reduced membranous E-cadherin and increased vimentin expression. We further demonstrated increased expression of EMT-related factors (ZEB1 and vimentin) and performed functional assays, including migration and spheroid formation (**Supplementary Figure S5**), which provided supporting evidence of CCL2-induced mesenchymal transition. Together, these changes provide morphological evidence supporting the role of CCL2 in promoting EMT-like phenotypic transitions in lung cancer cells. Although additional analyses such as invasion assays were not included, our findings are consistent with previous reports showing that CCL2 downregulated E-cadherin and upregulated vimentin (47–49), and enhanced migratory or invasive abilities in various cancer types, including hepatocellular carcinoma and bladder cancer (48, 50, 51). Together, these studies support the role of CCL2 as an EMT-promoting cytokine, and our results extend this concept to osimertinib-resistant lung cancer cells.

All these EMT-associated changes prompted further comparison with other mechanisms underlying osimertinib resistance. Recent studies have revealed multiple regulatory pathways contributing to osimertinib resistance, including those involved in epithelial–mesenchymal transition (EMT) and activation of alternative bypass signaling mechanisms. PIM1 kinase and RBM15 have recently been shown to promote EMT-associated osimertinib resistance through GSK3β/β-catenin activation and m6A-dependent silencing of SPOCK1 (52, 53), respectively. Clinically, MET amplification and EGFR C797S represent the most common acquired resistance mechanisms (54). Collectively, these findings highlight that both genetic and non-genetic events—such as MET amplification and EMT-related signaling—play critical roles in sustaining tumor survival.

Increasing evidence demonstrates that CCL2 is an essential component of the tumor microenvironment. The increased expression of CCL2 in the tumor microenvironment significantly impacts the effectiveness of trastuzumab (a HER2 monoclonal antibody) by promoting the recruitment of immunosuppressive cells, thereby dampening its anti-tumor effects in HER2-positive gastric cancer (55). Additionally, CCL2 plays a pivotal role in promoting hepatocellular carcinoma progression and sorafenib resistance by enhancing tumor cell survival, migration, and invasion (56, 57). Moreover, heightened CCL2 levels can counteract the anti-angiogenic effects of bevacizumab (a drug targeting vascular endothelial growth factor) in colorectal cancer (58). CCL2 plays a significant role in driving resistance to targeted therapies and facilitating tumor progression; however, its role in osimertinib-resistant lung cancer has not been thoroughly evaluated. In this study, we demonstrated that the increased expression of CCL2 contributes to the acquisition of osimertinib resistance in initially osimertinib-sensitive cells. Furthermore, knocking down CCL2 in osimertinib-resistant cells led to an enhancement of osimertinib responsiveness. Our research findings provide valuable insights into the significance of the CCL2-CCR2 signaling pathway in driving the development of resistance to a third-generation EGFR-TKI osimertinib in lung cancer.

Combining INCB3344 or CCL2 siRNA with osimertinib significantly suppressed cell survival. However, osimertinib-resistant cells exhibited residual levels of phosphorylated EGFR. Despite this, there may still be advantages to utilizing osimertinib in cell therapy. Treatment with osimertinib effectively inhibits phosphorylated EGFR and downstream phosphorylated AKT. In clinical practice, combinations of osimertinib with other targeted therapies have been explored in clinical trials involving patients who have experienced progression after osimertinib treatment. For example, James et al. reported the results of the phase 1b TATTON trial (Part B), assessing the efficacy of osimertinib plus selumetinib (MEK1/2 inhibitor) in EGFR mutant patients following EGFR TKI failure who were MET-negative. The study showed certain anti-tumor activities (ORR 34%, PFS 4.2 months) (59). Another example was the study of INSIGHT2: a phase II study of tepotinib plus osimertinib in MET-amplified NSCLC and first-line osimertinib resistance. The reported objective response rate (ORR) was 43.9% (95% CI 33.9, 54.3), median (m) DOR was 9.7 months (95% CI 5.6, ne), mPFS was 5.4 months (60).

Our study also emphasized the connection between CCL2-induced osimertinib resistance and the activation of the STAT3 and ERK signaling pathways. Our results indicated that both the STAT3 and ERK signaling pathways are downstream of CCL2 and are involved in CCL2-mediated osimertinib resistance in lung cancer. Activation of the ERK signaling pathway has been implicated in osimertinib resistance, as evidenced in our study using an osimertinib-resistant cell line model (**Figure 6**) and supported by the findings of Shi et al. (61) and Huang et al. (62). These findings suggest that the activation of the CCL2/ERK pathway facilitates the bypassing of EGFR inhibition induced by osimertinib. Additionally, our results indicated that CCL2 plays a role in the development of osimertinib resistance in lung cancer by activating the STAT3 pathway. Several studies have reported that cancer cells that acquire resistance to EGFR-TKIs (such as gefitinib, erlotinib, afatinib, and osimertinib) often display elevated levels of phosphorylated STAT3. Increased STAT3 activation is frequently associated with acquired resistance to EGFR-TKIs (63–66). Moreover, the findings presented by Sun et al. provide additional evidence supporting this idea, demonstrating that both STAT3 and IL-4 are involved in the development of osimertinib resistance (67). In the context of osimertinib resistance, activating the STAT3 signaling pathway confers a survival advantage to tumor cells by inhibiting cell death-associated pathways and orchestrating changes in the tumor microenvironment.

Multiple studies have elucidated the mechanistic relationship between CCL2 and the STAT3–ZEB1 signaling pathway. Specifically, binding of CCL2 to its receptor CCR2 induces conformational changes that facilitate the recruitment of Janus kinase 2 (JAK2) to the receptor complex (20). Activated JAK2 subsequently phosphorylates STAT3 at Tyr705, promoting STAT3 dimerization and nuclear translocation (68). Once translocated into the nucleus, STAT3 binds to the ZEB1 promoter, thereby repressing E-cadherin expression and promoting epithelial–mesenchymal transition, cell invasion, and drug resistance (69–71). In the present study, we provide direct experimental evidence supporting this mechanism. Chromatin immunoprecipitation (ChIP) analysis demonstrated significant enrichment of STAT3 at the ZEB1 promoter in osimertinib-resistant H1975/AZD-18 cells, establishing STAT3 as a direct transcriptional regulator of ZEB1 in this context. Together with our genetic and pharmacological inhibition data, these findings define a CCL2–CCR2–STAT3–ZEB1 signaling axis that drives EMT-associated osimertinib resistance. Furthermore, we demonstrated that both genetic and pharmacologic inhibition of CCR2 (INCB3344), STAT3 (S3I-201), or ERK (selumetinib) effectively disrupted CCL2-induced STAT3 phosphorylation and ZEB1 expression. Accordingly, targeting the CCL2–CCR2 signaling pathway enhanced osimertinib-induced cell death and reduced ZEB1 expression, supporting a central role for the STAT3/ERK–ZEB1 axis in mediating CCL2-mediated resistance to EGFR-TKIs in lung cancer.

In addition to genetic and small-molecule inhibition strategies, we further evaluated the functional contribution of extracellular CCL2 using a neutralizing antibody approach. Treatment with carlumab, significantly enhanced osimertinib-induced cytotoxicity in osimertinib-resistant H1975/AZD-18 cells. This finding provides direct functional evidence that secreted CCL2 acts as an extracellular regulator of osimertinib resistance, rather than merely reflecting a correlative biomarker.

However, this study has certain methodological limitations. Genetic rescue experiments using constitutively active STAT3 in CCL2-knockdown-resistant cells to examine ZEB1 mRNA expression were not performed. Complementary analyses, such as quantitative pathway activity profiling were not included. Although S3I-201 and INCB3344 effectively inhibited STAT3 and CCR2 signaling, respectively, both compounds may have off-target effects. Therefore, future studies should incorporate genetic approaches to further validate and strengthen these findings.

The activated states of STAT3 and ERK are implicated in maintaining osimertinib resistance. Consequently, it was anticipated that the STAT3 inhibitor S3I201 would exert an intermediate inhibitory effect on tumor volume compared to the untreated and combination groups (**Figure 6I**). However, experimental results revealed that S3I201 did not induce tumor shrinkage, possibly attributable to higher IL-6 mRNA expression in osimertinib-resistant cells (data not shown). IL-6, induced by NF-κB or STAT3, can in turn feedback to activate STAT3 and NF-κB (72, 73). The NF-κB activation pathway, engaged in response to EGFR oncogene inhibition, promotes tumor cell survival and drives resistance to EGFR TKIs (74–76). Through their functional interaction, NF-κB and STAT3 collaboratively contribute to osimertinib resistance.

ZEB1, a transcription factor linked to the epithelial-mesenchymal transition (EMT), plays a crucial role in enhancing cancer cell invasiveness and confers resistance to targeted therapies (77, 78). Previous studies have indicated that CD44 promotes lung cancer cell metastasis and may potentially contribute to epithelial-mesenchymal transition (EMT) by activating the ERK-ZEB1 signaling pathway (79). Following treatment with EGFR inhibitors, the increased expression of ZEB1 leads to alterations in cellular behavior, resulting in reduced reliance on EGFR signaling pathways for survival (78). ZEB1-mediated EMT has been identified as one of the underlying causes of erlotinib resistance in EGFR-mutant lung cancer cells (77). Moreover, the activation of the STAT3-ZEB1 signaling pathway enhance EGFR-TKI resistance in lung cancer (80). Indeed, both the ERK-ZEB1 and STAT3-ZEB1 axes are interconnected and have a significant impact on cancer progression and drug resistance. The alterations in cell behavior induced by EMT contribute to reduced sensitivity to a variety of targeted therapies. Our findings reveal that CCL2-mediated osimertinib resistance in lung cancer through up-regulating ZEB1 expression. Knockdown of ZEB1 expression in CCL2-overexpressing transfectants significantly suppressed the CCL2-mediated osimertinib resistance. In this study, we also indicated that CCL2 upregulated ZEB1 expression in lung cancer cells through activating STAT3 and ERK signaling pathways. The combination treatment of the STAT3 inhibitor (S3I201) or ERK inhibitor (selumetinib) with osimertinib in CCL2-mediated osimertinib resistance cells leads to the suppression of CCL2-induced ZEB1 expression and the subsequent reversal of CCL2-mediated resistance to osimertinib.

Importantly, this study offers several distinctive advances compared with previous CCL2-related reports in other cancers. we identified and validated a previously unrecognized STAT3/ERK–ZEB1 signaling axis that mediates to CCL2-driven resistance to EGFR-TKIs in lung cancer, rather than acting through the AKT-dependent pathways described in earlier studies. Our findings were verified using patient-derived malignant pleural effusions, and *in vivo* xenografts, providing strong translational relevance. Together, these findings extend the understanding of CCL2 biology and uncover a new therapeutic target to overcome osimertinib resistance in NSCLC.

## 5 Conclusion

Our study has demonstrated that the CCL2-CCR2 signaling axis triggers increased phosphorylation of STAT3 and ERK, subsequently leading to enhance ZEB1 expression in lung cancer. It is noteworthy that the CCL2-CCR2 pathway not only contributes to osimertinib resistance but also actively participates in cancer progression. To conclude, our findings strongly support the pivotal role of CCL2 in the development of acquired osimertinib resistance in lung cancer. Targeting the CCL2-CCR2-STAT3/ERK-ZEB1 signaling pathway emerges as a promising therapeutic approach for overcoming osimertinib resistance and enhancing treatment outcomes in this context. Overall, this study addresses a significant and timely question, and the proposed pathway has potential clinical relevance.

## Data Availability

The original contributions presented in the study are included in the article/**Supplementary Material**. Further inquiries can be directed to the corresponding author.
