# Supplementary material for: The STAT3–ZEB1 axis contributes to CCL2-mediated resistance to osimertinib in lung cancer
Source: Front Oncol. 2026 Feb 20;16:1699471. doi: 10.3389/fonc.2026.1699471 (PMC12962936; doi:10.3389/fonc.2026.1699471)
Supplement: Supplementary file 1 [file DataSheet1.docx]

**Supplementary methods**

**Establishment of shCCL2 and shZEB1 Stable Cell Lines**

Lentiviral shRNA particles targeting CCL2 (shCCL2), ZEB1 (shZEB1) and a non-targeting control (shCtrl) were obtained from the National RNAi Core Facility (Academia Sinica, Taiwan). Cells were transduced with lentiviral particles at a multiplicity of infection (MOI) of 5 in complete RPMI 1640 supplemented with 8 μg/mL polybrene for 24 hours. Subsequently, cells were subjected to Puromycin selection for 96 hours following the manufacturer's protocol.

**Chemokine Detection**

To detect the chemokines secreted by osimertinib-resistant cells, we utilized the Proteome Profiler™ Human Chemokine Array Kit (R&D Systems, ARY017), following the manufacturer's instructions. Briefly, array membranes were blocked for 1 hour and then incubated overnight with conditioned medium collected from cells cultured at a density of 2 x 10^5^ cells/mL for 24 hours. Subsequently, the membranes were washed and incubated with horseradish peroxidase (HRP)-conjugated streptavidin in blocking buffer.

**Immunofluorescence staining**

Cells were seeded on Millicell EZ SLIDE 4-well chambers (PEZGS0416, Merck Millipore, Darmstadt, Germany) and cultured for 24 h. Cells were fixed with 4% paraformaldehyde at room temperature for 10 min, permeabilized with 0.1% Triton X-100 in PBS, and blocked with 1% bovine serum albumin for 1 h. Cells were then incubated overnight at 4 °C with mouse anti-E-cadherin (610181, BD; 1:400) or rabbit anti-vimentin (#5741, Cell Signaling Technology; 1:200). After washing, cells were incubated with Alexa Fluor 647-conjugated goat anti-mouse IgG (#A-21236) or Alexa Fluor Plus 555-conjugated goat anti-rabbit IgG secondary antibodies (#A-21429; Invitrogen; 1:1000) for 45 min at room temperature. Nuclei were counterstained with DAPI, and images were acquired using a Leica SP8X confocal microscope.

**Migration assay**

Transwell migration assays were carried out using 8-μm pore inserts (Costar, Corning). Briefly, 1.5 × 10⁴ cells were plated in the upper chamber and incubated for 18 h. Non-migrated cells were removed with a cotton swab, and migrated cells on the lower surface were fixed with methanol, stained with crystal violet, and counted using an EVOS imaging system (Thermo Fisher Scientific).

**Sphere formation**

H1975 cells treated with or without 5 ng/mL recombinant human CCL2 were seeded (5,000 cells/well) in 24-well ultra-low attachment plates (Corning). Cells were cultured in serum-free DMEM/F12 medium (1:1, Gibco) supplemented with B27, HEPES, bFGF, EGF, ITS, and antibiotics under standard conditions (37 °C, 5% CO₂). Growth factors were replenished every 2 days. After 7 days, the number and diameter of spheres were measured, and representative images were acquired using an EVOS imaging system (Thermo Fisher Scientific).

**Drug interaction analysis**

Cell viability was assessed as described in the cytotoxicity assays section of the manuscript. To quantify the interactions between osimertinib and INCB3344/S3I201, SynergyFinder (https://synergyfinder.fimm.fi/synergy/20240807100232180928/) was used to estimate the synergy scores of the proposed combination therapy. Synergy scores were calculated based on the zero interaction potency (ZIP) reference model [1].

**Supplementary Tables**

**Table S1.** A**ntibodies used for Western blot in the study**

| Name | Catalog | Company | Dilution factor | Application |
| --- | --- | --- | --- | --- |
| ZEB1 | #70512 | Cell signaling | 1:1000 | WB |
| SNAI1 | #3879 | Cell signaling | 1:800 | WB |
| p-EGFR (Tyr1068) | #2236 | Cell signaling | 1:1000 | WB |
| EGFR | SC-03 | Santa cruz | 1:1500 | WB |
| p-AKT(Ser473) | #4060 | Cell signaling | 1:1500 | WB |
| AKT | #9272 | Cell signaling | 1:2000 | WB |
| p-ERK1/2 (Thr202/Tyr204) | #9101 | Cell signaling | 1:2000 | WB |
| ERK1/2 | #9102 | Cell signaling | 1:2500 | WB |
| p-STAT3 (Tyr705) | GTX61820 | GeneTex | 1:2000 | WB |
| STAT3 | #4904 | Cell signaling | 1:2000 | WB |
| CCR2 | #12199 | Cell signaling | 1:1500 | WB |
| Slug | #9585 | Cell signaling | 1:1000 | WB |
| PARP | #9532 | Cell signaling | 1:1500 | WB |
| E-cadherin | #3195 | Cell signaling | 1:2000 | WB |
| vimentin | #5741S | Cell Signaling | 1:2500 | WB |
| α-tubulin | #05-829 | Millipore | 1:7500 | WB |

**Table S2. Primers used for quantitative real-time PCR**

| Gene names | Forward | Reverse |
| --- | --- | --- |
| TBP | 5′-ACGCCAGCTTCGGAGAGTT-3′ | 5′-CCTCATGATTACCGCAGCAAA-3′ |
| CCL2 | 5′-AAGATCTCAGTGCAGAGGCTCG-3′ | 5′-TTGCTTGTCCAGGTGGTCCAT-3′ |
| ZEB1 | 5′-TGACAGAAAGGAAGGGCAAGA-3′ | 5′-CAGGTGAGTAATTGTGAAAATGCATGT-3′ |
| ZEB1-P | 5′-AGCCGATGCTTCTTGCCTTAA-3′ | 5′-TAAGGTATCCACAGGCCATGAA-3′ |

**Table S3. Top 100 EGFR TKI resistance-related genes.**

| Rank | gene_id | gene_name | gene_type | score |
| --- | --- | --- | --- | --- |
| 1 | ENSG00000163453 | IGFBP7 | protein_coding | 3.85 |
| 2 | ENSG00000129965 | INS-IGF2 | protein_coding | 3.64 |
| 3 | ENSG00000148677 | ANKRD1 | protein_coding | 3.48 |
| 4 | ENSG00000143546 | S100A8 | protein_coding | 3.45 |
| 5 | ENSG00000118523 | CTGF | protein_coding | 3.42 |
| 6 | ENSG00000207279 | SNORD116-24 | snoRNA | 3.37 |
| 7 | ENSG00000164379 | FOXQ1 | protein_coding | 3.23 |
| 8 | ENSG00000151892 | GFRA1 | protein_coding | 3.18 |
| 9 | ENSG00000115461 | IGFBP5 | protein_coding | 3.10 |
| 10 | ENSG00000119714 | GPR68 | protein_coding | 2.69 |
| 11 | ENSG00000165092 | ALDH1A1 | protein_coding | 2.65 |
| 12 | ENSG00000145934 | TENM2 | protein_coding | 2.57 |
| 13 | ENSG00000155622 | XAGE2 | protein_coding | 2.55 |
| 14 | ENSG00000138131 | LOXL4 | protein_coding | 2.40 |
| 15 | ENSG00000168386 | FILIP1L | protein_coding | 2.40 |
| 16 | ENSG00000130176 | CNN1 | protein_coding | 2.36 |
| 17 | ENSG00000108691 | CCL2 | protein_coding | 2.30 |
| 18 | ENSG00000230043 | TMSB4XP6 | processed_pseudogene | 2.24 |
| 19 | ENSG00000175984 | DENND2C | protein_coding | 2.16 |
| 20 | ENSG00000153707 | PTPRD | protein_coding | 2.13 |
| 21 | ENSG00000078401 | EDN1 | protein_coding | 2.10 |
| 22 | ENSG00000115008 | IL1A | protein_coding | 2.08 |
| 23 | ENSG00000123610 | TNFAIP6 | protein_coding | 2.07 |
| 24 | ENSG00000244067 | GSTA2 | protein_coding | 2.02 |
| 25 | ENSG00000175928 | LRRN1 | protein_coding | 2.01 |
| 26 | ENSG00000213626 | LBH | protein_coding | 1.98 |
| 27 | ENSG00000106366 | SERPINE1 | protein_coding | 1.97 |
| 28 | ENSG00000125538 | IL1B | protein_coding | 1.94 |
| 29 | ENSG00000260604 | AL590004.4 | lincRNA | 1.93 |
| 30 | ENSG00000077782 | FGFR1 | protein_coding | 1.93 |
| 31 | ENSG00000076716 | GPC4 | protein_coding | 1.91 |
| 32 | ENSG00000144476 | ACKR3 | protein_coding | 1.90 |
| 33 | ENSG00000125740 | FOSB | protein_coding | 1.89 |
| 34 | ENSG00000073756 | PTGS2 | protein_coding | 1.88 |
| 35 | ENSG00000207174 | SNORD116-15 | snoRNA | 1.85 |
| 36 | ENSG00000101335 | MYL9 | protein_coding | 1.85 |
| 37 | ENSG00000184500 | PROS1 | protein_coding | 1.84 |
| 38 | ENSG00000206621 | SNORD116-14 | snoRNA | 1.83 |
| 39 | ENSG00000173706 | HEG1 | protein_coding | 1.82 |
| 40 | ENSG00000118785 | SPP1 | protein_coding | 1.82 |
| 41 | ENSG00000134258 | VTCN1 | protein_coding | 1.81 |
| 42 | ENSG00000117114 | ADGRL2 | protein_coding | 1.79 |
| 43 | ENSG00000112299 | VNN1 | protein_coding | 1.76 |
| 44 | ENSG00000117020 | AKT3 | protein_coding | 1.76 |
| 45 | ENSG00000128422 | KRT17 | protein_coding | 1.73 |
| 46 | ENSG00000173391 | OLR1 | protein_coding | 1.73 |
| 47 | ENSG00000091986 | CCDC80 | protein_coding | 1.72 |
| 48 | ENSG00000153071 | DAB2 | protein_coding | 1.72 |
| 49 | ENSG00000108342 | CSF3 | protein_coding | 1.71 |
| 50 | ENSG00000115009 | CCL20 | protein_coding | 1.70 |
| 51 | ENSG00000164125 | FAM198B | protein_coding | 1.69 |
| 52 | ENSG00000163734 | CXCL3 | protein_coding | 1.69 |
| 53 | ENSG00000261040 | WFDC21P | processed_transcript | 1.69 |
| 54 | ENSG00000207014 | SNORD116-3 | snoRNA | 1.68 |
| 55 | ENSG00000165685 | TMEM52B | protein_coding | 1.68 |
| 56 | ENSG00000124102 | PI3 | protein_coding | 1.67 |
| 57 | ENSG00000180537 | RNF182 | protein_coding | 1.67 |
| 58 | ENSG00000163283 | ALPP | protein_coding | 1.66 |
| 59 | ENSG00000122641 | INHBA | protein_coding | 1.65 |
| 60 | ENSG00000210077 | MT-TV | Mt_tRNA | 1.64 |
| 61 | ENSG00000170390 | DCLK2 | protein_coding | 1.64 |
| 62 | ENSG00000186340 | THBS2 | protein_coding | 1.63 |
| 63 | ENSG00000207093 | SNORD116-8 | snoRNA | 1.61 |
| 64 | ENSG00000137801 | THBS1 | protein_coding | 1.61 |
| 65 | ENSG00000183688 | RFLNB | protein_coding | 1.59 |
| 66 | ENSG00000182326 | C1S | protein_coding | 1.59 |
| 67 | ENSG00000169429 | CXCL8 | protein_coding | 1.55 |
| 68 | ENSG00000166250 | CLMP | protein_coding | 1.54 |
| 69 | ENSG00000109511 | ANXA10 | protein_coding | 1.54 |
| 70 | ENSG00000081052 | COL4A4 | protein_coding | 1.53 |
| 71 | ENSG00000176907 | C8orf4 | protein_coding | 1.52 |
| 72 | ENSG00000144810 | COL8A1 | protein_coding | 1.52 |
| 73 | ENSG00000236091 | LINC02243 | lincRNA | 1.51 |
| 74 | ENSG00000243955 | GSTA1 | protein_coding | 1.50 |
| 75 | ENSG00000137393 | RNF144B | protein_coding | 1.50 |
| 76 | ENSG00000172575 | RASGRP1 | protein_coding | 1.49 |
| 77 | ENSG00000158186 | MRAS | protein_coding | 1.48 |
| 78 | ENSG00000207001 | SNORD116-2 | snoRNA | 1.48 |
| 79 | ENSG00000120129 | DUSP1 | protein_coding | 1.48 |
| 80 | ENSG00000170558 | CDH2 | protein_coding | 1.48 |
| 81 | ENSG00000207442 | SNORD116-6 | snoRNA | 1.48 |
| 82 | ENSG00000231389 | HLA-DPA1 | protein_coding | 1.48 |
| 83 | ENSG00000142871 | CYR61 | protein_coding | 1.48 |
| 84 | ENSG00000131459 | GFPT2 | protein_coding | 1.48 |
| 85 | ENSG00000134668 | SPOCD1 | protein_coding | 1.47 |
| 86 | ENSG00000167601 | AXL | protein_coding | 1.46 |
| 87 | ENSG00000085563 | ABCB1 | protein_coding | 1.46 |
| 88 | ENSG00000165376 | CLDN2 | protein_coding | 1.46 |
| 89 | ENSG00000176046 | NUPR1 | protein_coding | 1.46 |
| 90 | ENSG00000166741 | NNMT | protein_coding | 1.45 |
| 91 | ENSG00000131378 | RFTN1 | protein_coding | 1.45 |
| 92 | ENSG00000111305 | GSG1 | protein_coding | 1.45 |
| 93 | ENSG00000131016 | AKAP12 | protein_coding | 1.44 |
| 94 | ENSG00000086289 | EPDR1 | protein_coding | 1.44 |
| 95 | ENSG00000139278 | GLIPR1 | protein_coding | 1.44 |
| 96 | ENSG00000130433 | CACNG6 | protein_coding | 1.43 |
| 97 | ENSG00000166033 | HTRA1 | protein_coding | 1.43 |
| 98 | ENSG00000171711 | DEFB4A | protein_coding | 1.42 |
| 99 | ENSG00000185275 | CD24P4 | processed_pseudogene | 1.42 |
| 100 | ENSG00000100100 | PIK3IP1 | protein_coding | 1.42 |

**Table S4.** The pleural effusion from the 63 patients with advanced lung adenocarcinoma who received EGFR-TKI therapies

|  |  | **Treatment-naive** | **Acquired resistance to osimertinib** | **Acquired resistance to 1^st^/2^nd^-G TKIs^*^** | ***p-value*** |
| --- | --- | --- | --- | --- | --- |
| **Total No.** | | 26 | 16 | 21 |  |
| **Age, median years**  **(range)** | | 66.4  (43.5–85.2) | 73.9  (40.1–90.0) | 63.6  (29.7–91.4) | 0. 063^#^ |
| **Sex** | |  |  |  | 0.906 |
|  | **Female** | 17 | 11 | 15 |  |
|  | **Male** | 9 | 5 | 6 |  |
| **Smoking** | |  |  |  | 0.665 |
|  | **Nonsmokers** | 22 | 15 | 18 |  |
|  | **Smokers** | 4 | 1 | 3 |  |
| ***EGFR* mutation** | |  |  |  | 0.478 |
|  | **Del-19** | 14 | 11 | 14 |  |
|  | **L858R** | 11 | 5 | 5 |  |
|  | **Other** | 1 | 0 | 2 |  |

^#^ By Kruskal–Wallis test; **^*^** 1^st^/2^nd^-G TKIs: 1^st^- or 2^nd^-generation EGFR TKIs

Del-19: Deletion in exon 19; EGFR TKI: Epidermal growth factor receptor tyrosine kinase inhibitor

**Supplementary Figures**

**Fig. S1** (A) Raw blots of chemokine arrays showing cytokines secreted by HCC827 and HCC827/gef cells. The Raw blots are highlighted within red boxes. (B) Quantification of relative spot intensity compared to the control blot of HCC827 using ImageJ (***p<0.001). (C) Raw blots of chemokine arrays displaying cytokines secreted by H1975, H1975/AZD-15, and H1975/AZD-18 cells. (D) Quantification of relative spot intensity compared to the control blot of H1975 using Image J (***p<0.001).


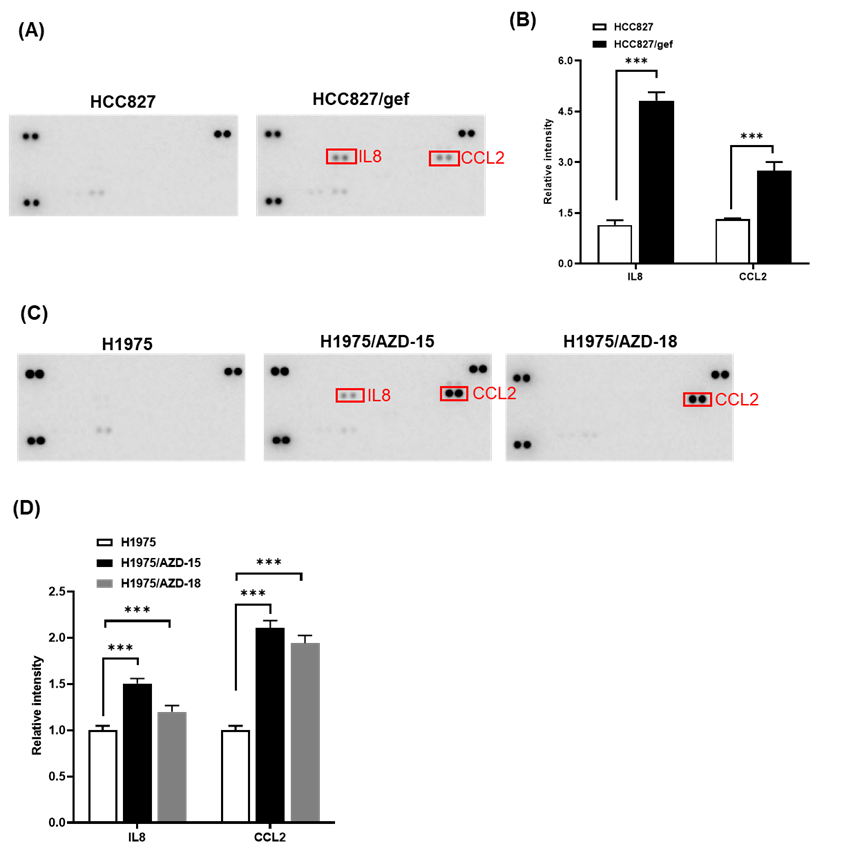


**Fig.** **S2** (A, C, and E) CCL2 expression levels were assessed by RT-qPCR (left) and Western blot analysis (right) in cells stably knocked down for CCL2 using CCL2 shRNA, along with corresponding control transfectants (*** p < 0.001). (B, D, and F) Cellular viability of CCL2-knockdown and control stable cells was evaluated after 72-hour treatment with varying doses of osimertinib using the MTT assay (*** p < 0.001).

**Fig. S3** The survival curve of CCL2 in lung cancer patients based on K-M plotter database (http://www.kmplot.com). (p < 0.05)

**
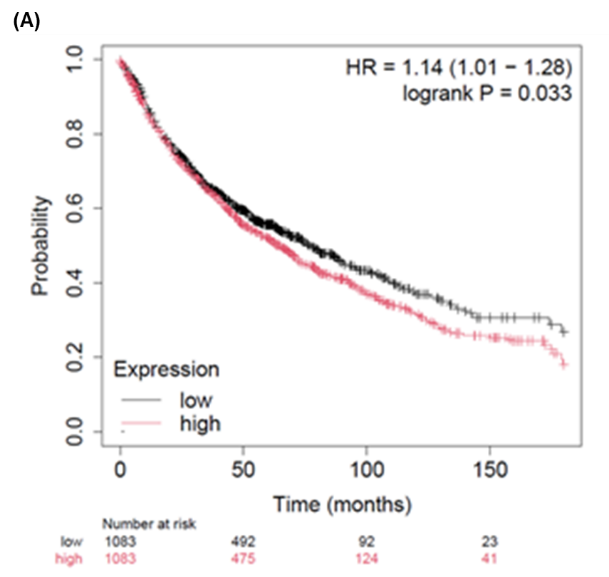
**

**Fig. S4 (A)**The expression levels of EMT regulators (ZEB1, SNAI1, and Slug) and vimentin (VIM) were assessed in HCC827-CCL2 control knockdown transfectants and CCL2 knockdown transfectants by utilizing CCL2 shRNA, followed by Western blot analysis. HCC827/gef-shCtrl and H1975/AZD-18-shCtrl were the control groups. (B) Immunofluorescence analysis of EMT markers in CCL2-overexpressing lung cancer cells. HCC827-mock and HCC827-CCL2 cells were stained for vimentin (VIM, red) and E-cadherin (E-cad, green), with nuclei counterstained using DAPI (blue). Scale bars = 50 μm.


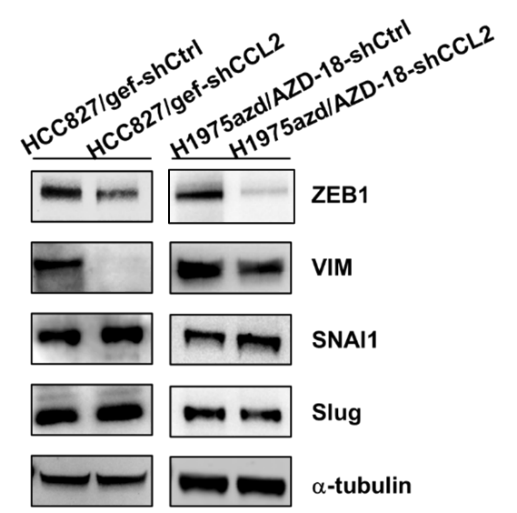


**(B)**

**(A)**

**Fig. S5** (A) Representative images of the migration assay performed using HCC827-CCL2 and control cells (upper panel). Cells that migrated through the membrane were fixed, stained, and counted after 18 h. Quantification of migrated cells is shown in the lower panel. Data represent the mean ± SD from three independent experiments (***p < 0.001, Student’s t-test). (B) Representative images of the spheroid formation assay in H1975 cells treated without or with 5 ng/ml recombinant human CCL2 (rCCL2). The number and diameter of spheroids were quantified after 7 days of culture under non-adherent conditions. Data represent the mean ± SD from three independent experiments (**p < 0.01, Student’s t-test).


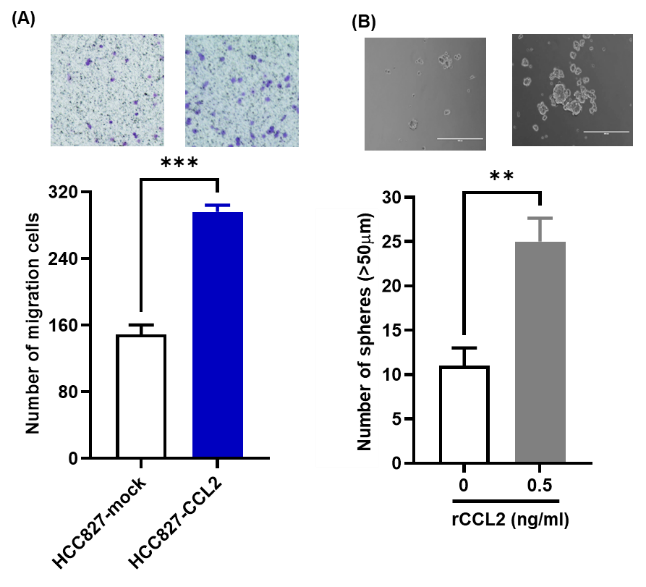


**Fig. S6** (A) HCC827-CCL2 cells were subjected to ZEB1 knockdown using shRNA, and the expression levels of ZEB1 mRNA and protein were assessed via RT-qPCR and Western blotting (***p<0.001). (B) The cellular viability of stable cells with ZEB1 knockdown and control was evaluated after treatment with various doses of osimertinib for 48 hours using MTT assays (n = 3 independent experiments, ***p<0.001)


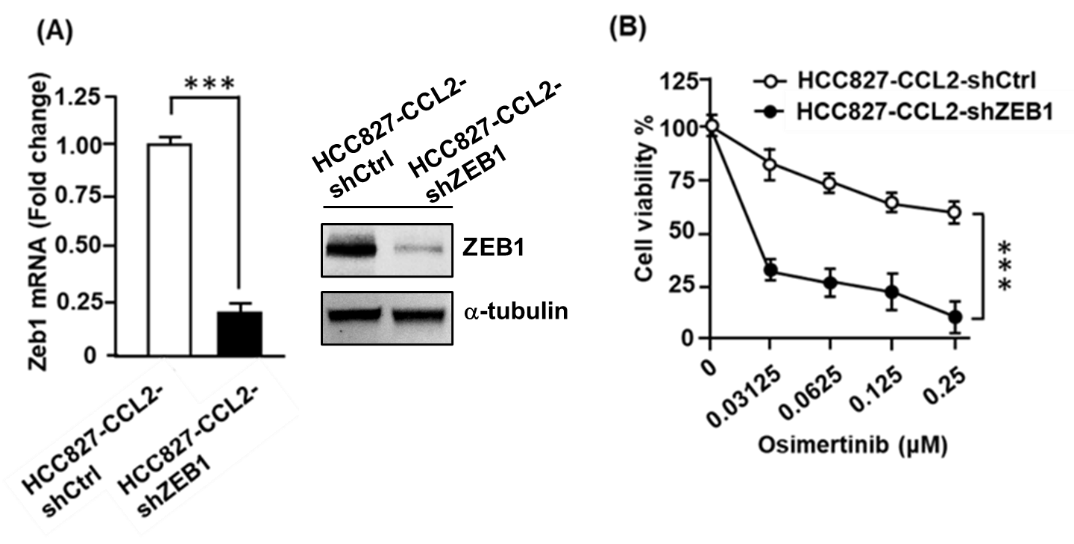


**Fig. S****7 (**A) ZEB1 and VIM expression levels were assessed in HCC827-CCL2 cells using pool si-ZEB1 and pool siVIM, followed by Western blot analysis. (B) Cellular viability of ZEB1-knockdown, vimentin-knockdown, and control transfectants was determined after treatment with various doses of osimertinib for 48 hours using the MTT assay. Error bars represent standard deviations for n = 3 independent experiments (** p < 0.01, *** p < 0.001, ns: not statistically significant).


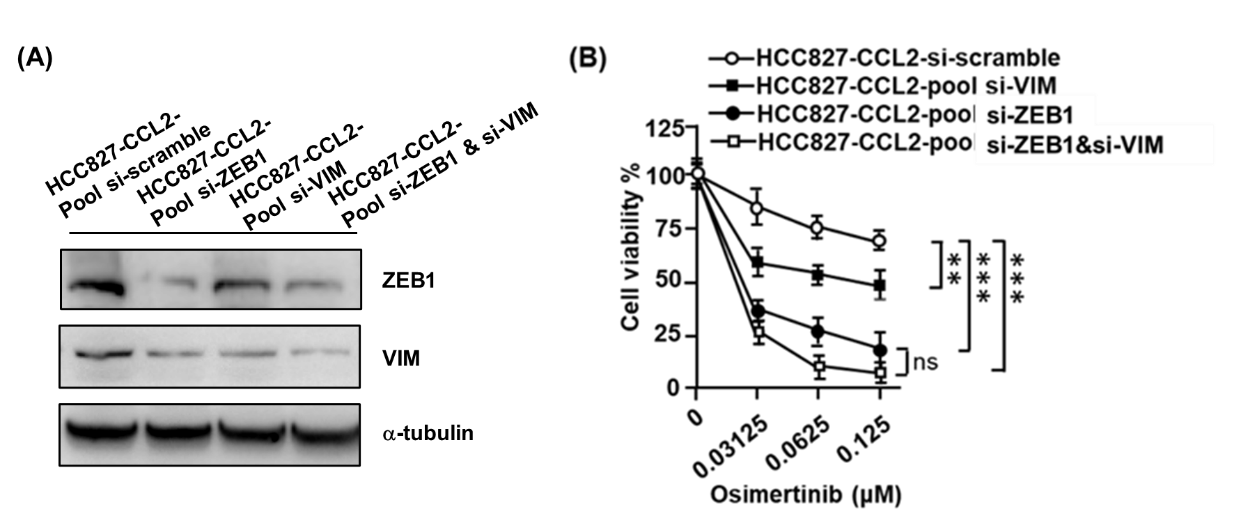


**Fig. S8** (A) HCC827-CCL2 cells were treated with increasing concentrations of osimertinib and the CCR2 antagonist INCB3344 for 48h. Cell viability was assessed using an MTT assay, and the drug combination responses was analyzed using Synergy Finder. The 3D map of the ZIP synergy score shows a mean score of 23.15, indicating a synergistic interaction. (B) The 3D map of the ZIP synergy score for HCC827-CCL2 cells treated with osimertinib and S3I201 shows a score of 28.012, indicating a synergistic interaction. ZIP Scores > 10 were considered to indicate synergistic effects.

**
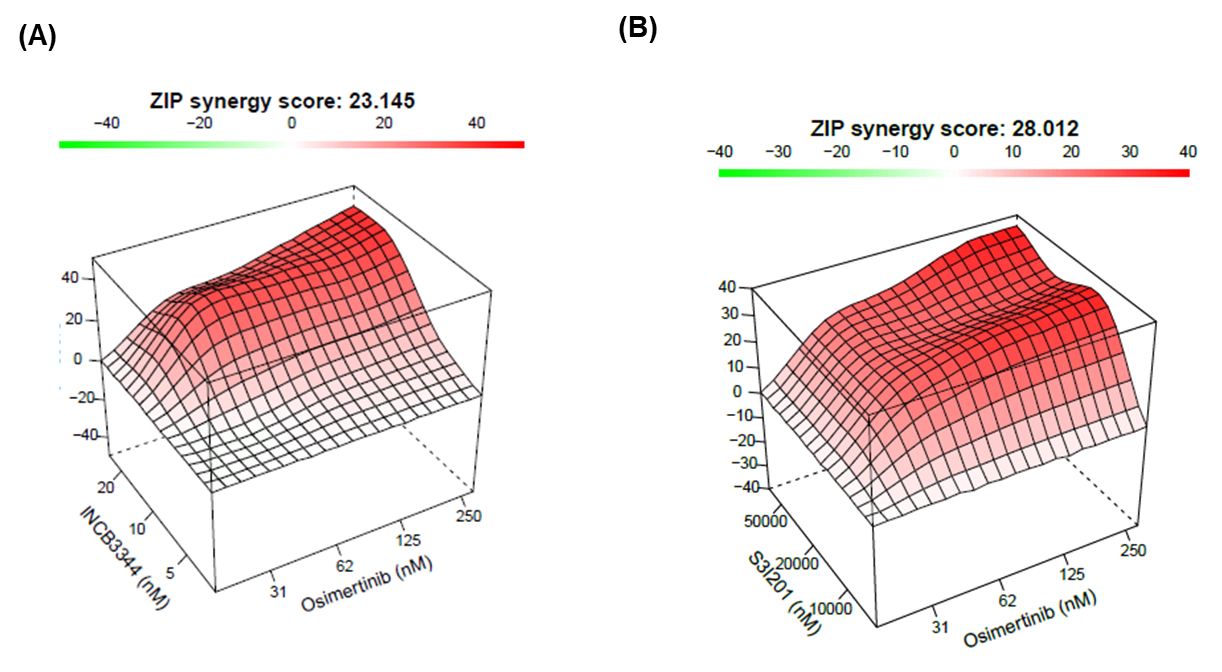
**

**References**

1. Ianevski A, Giri AK & Aittokallio T (2020) SynergyFinder 2.0: visual analytics of multi-drug combination synergies. Nucleic Acids Res 48, W488-w493, doi: 10.1093/nar/gkaa216.
